# Surface Functionalisation of Dental Implants with a Composite Coating of Alendronate and Hydrolysed Collagen: DFT and EIS Studies

**DOI:** 10.3390/ma15155127

**Published:** 2022-07-23

**Authors:** Željka Petrović, Ankica Šarić, Ines Despotović, Jozefina Katić, Robert Peter, Mladen Petravić, Mile Ivanda, Marin Petković

**Affiliations:** 1 Division of Materials Chemistry, Ruđer Bošković Institute, Bijenička cesta 54, 10002 Zagreb, Croatia; 2 Division of Materials Physics, Ruđer Bošković Institute, Bijenička cesta 54, 10002 Zagreb, Croatia; ivanda@irb.hr; 3 Centre of Excellence for Advanced Materials and Sensing Device, Ruđer Bošković Institute, Bijenička cesta 54, 10002 Zagreb, Croatia; 4 Division of Physical Chemistry, Ruđer Bošković Institute, Bijenička cesta 54, 10002 Zagreb, Croatia; ines.despotovic@irb.hr; 5 Department of Electrochemistry, Faculty of Chemical Engineering and Technology, University of Zagreb, Marulićev trg 19, 10000 Zagreb, Croatia; jkatic@fkit.hr; 6 Faculty of Physics and Center for Micro- and Nanosciences and Technologies, University of Rijeka, R. Matejcic 2, 51000 Rijeka, Croatia; rpeter@phy.uniri.hr (R.P.); mpetravic@phy.uniri.hr (M.P.); 7 Poliklinika Petković, Lašćinska cesta 97, 10000 Zagreb, Croatia; info@poliklinika-petkovic.hr

**Keywords:** titanium dental implant, sodium alendronate, hydrolysed collagen, functionalisation, DFT, XPS, EIS

## Abstract

The success of the osseointegration process depends on the surface characteristics and chemical composition of dental implants. Therefore, the titanium dental implant was functionalised with a composite coating of alendronate and hydrolysed collagen, which are molecules with a positive influence on the bone formation. The results of the quantum chemical calculations at the density functional theory level confirm a spontaneous formation of the composite coating on the titanium implant, ∆G*_INT_ = −8.25 kcal mol^−1^. The combination of the results of X-ray photoelectron spectroscopy and quantum chemical calculations reveals the structure of the coating. The alendronate molecules dominate in the outer part, while collagen tripeptides prevail in the inner part of the coating. The electrochemical stability and resistivity of the implant modified with the composite coating in a contact with the saliva depend on the chemical nature of alendronate and collagen molecules, as well as their inter- and intramolecular interactions. The formed composite coating provides a 98% protection to the implant after the 7-day immersion in the artificial saliva. From an application point of view, the composite coating could effectively promote osseointegration and improve the implant’s resistivity in contact with an aggressive environment such as saliva.

## 1. Introduction

The surface chemistry and properties are key factors of the implant’s long life since the surface of the implant is in direct contact with surrounding bones of the oral cavity [1]. Therefore, many commercially available implants made of metals, alloys, and ceramics have been intensively explored to evaluate their surface properties [2,3,4]. The results revealed the presence of inorganic and organic contaminants on many implants. To ensure appropriate implant’s surface characteristics, manufacturers use various surface treatments and processes such as high-temperature acid etching, anodising, sand and grit blasting, plasma spraying, or surface polishing [5,6,7,8]. For example, some studies have shown that rough implant surfaces favour the proliferation of bone-forming cells, or osteoblasts [7,8,9], which is why surface treatment with Al_2_O_3_ particles as abrasive agents is commonly used. Although the implants meet very strict international quality standards, Al_2_O_3_ particles and other contaminants can remain present on the implant’s surface.

It must not be forgotten that a human body, especially the oral cavity, is an aggressive environment containing different ions, enzymes, bacteria, and frequent daily pH changes. Such an environment can cause the release of contaminants from the implant’s surface into surrounding tissues and organs, which can trigger negative biological processes such as allergies or inflammation [10]. All this clearly underlines the need for improving the production process and introducing regular strict quality controls of implants. The chemical functionalisation of the implant’s surfaces is a simple way of improvement of surface properties, as well as resistivity of the implant during an exposure to the aggressive environment [1]. Biomimetic coatings [11] and coatings based on compounds with a positive effect on the bone system [12] have been in the focus of research in recent years. These coatings will behave as a barrier between the implant and the surrounding media, which can stop the release of contaminants from the implant into tissues and organs [13].

This study was focused on the titanium dental implant with detected aluminium (7 at.%). The main goal was to create a coating, which can improve overall resistivity of the implant during an exposure to the artificial saliva. Molecules of alendronate, a drug for bone diseases [12], and hydrolysed collagen, a biopolymer that provides structural and mechanical support to bones and connective tissues [14], were used for the functionalisation of the implant. A combination of results of X-ray photoelectron spectroscopy (XPS) and quantum chemical calculations at the density functional theory level (DFT) enabled a determination of a complex formation mechanism of the coatings. The influence of the coatings on overall electrochemical stability of the implants was explored by impedance spectroscopy (EIS) in the artificial saliva.

## 2. Materials and Methods

### 2.1. Chemicals, Solutions, and Materials

Solutions of hydrolysed collagen (Medex d.o.o., Slovenia) and sodium alendronate trihydrate (Merck Sharp & Dohme, Kenilworth, NJ, USA) were used to functionalise implants surfaces. The powders were dissolved in Milli-Q^®^ water (Millipore, Merck, Darmstadt, Germany) to prepare a 10 mmol dm^−3^ solution of each compound. A mixed solution of hydrolysed collagen and alendronate was prepared as follows. The powder of the hydrolysed collagen was dissolved ultrasonically (*f* = 35 kHz; 10 min) in the alendronate solution (10 mmol dm^−3^). The final concentration of the collagen solution was 10 mmol dm^−3^.

Grade 2 titanium dental implants (Ankylos^®^ C/X A11, Dentsply Friadent^®^ GmbH, Baden-Wuerttemberg, Germany) [15] were used as substrates for surface functionalisation.

### 2.2. Functionalisation of the Implant Surfaces

Before coatings preparation, the surfaces of the as-received implants were ultrasonically degreased with acetone (p.a., Gram-Mol, Zagreb, Croatia) and absolute ethanol (p.a., Gram-Mol, Croatia). The samples were rinsed with Milli-Q^®^ water, dried in a nitrogen stream (99.999%, Messer, Bad Soden, Germany), and immersed immediately in the prepared solutions at 22 ± 2 °C for 24 h. To ensure the chemical stability of the coatings on the implant’s surfaces, the modified samples were dried at 70 °C for 7 h after removal from the solutions [13,16]. Then, they were rinsed with Milli-Q^®^ water and absolute ethanol and dried in a nitrogen stream. To evaluate the influence of the composite coating on the properties of the implant, it was necessary to investigate the influence of each composite component. Therefore, the implant surfaces were functionalised with alendronate, hydrolysed collagen, and composite coatings.

### 2.3. Characterisation of the Implants

The morphological characteristics and elemental analysis of the implant surfaces were studied by the field emission scanning electron microscope (SEM, model JSM-7000F, Jeol Ltd., Tokyo, Japan) in conjunction with the Oxford Instruments energy dispersive X-ray analyser EDS/INCA 350 at 10 kV.

The Raman spectra were recorded in the T64000 (HORIBA Jobin Yvon, Kyoto, Japan) triple Raman spectrometer with a 532 nm diode laser. The laser excitation power was 5 mW.

The attenuated total reflection Fourier transform infrared (ATR-FTIR) spectra were measured by the Frontier spectrometer (PerkinElmer, Waltham, MA, USA) from 4000 to 370 cm^−1^ with a resolution of 4 cm^−1^ and 16 scans per measurement. The results shown represent the average of three measurements.

The X-ray photoelectron spectroscopy (XPS) analysis was carried out in the SPECS instrument, using monochromatised Al Kα line of 1486.74 eV. For the measurements around Ti 2*p*, O 1*s*, and C 1*s* core levels, the pass energy of the electron energy analyser (Phoibos MCD 100) was set to 10 eV, while the pass energy of 20 eV was used for the measurements around N 1*s* core level. The experimental spectra were fitted with the product of Gaussian and Lorentzian functions with Shirley background subtraction [17]. The binding energy (BE) of all photoemission spectra was calibrated by the BE of the C 1*s* peak at 285.0 eV.

The electrochemical behaviour of the implants was investigated in a three-electrode cell (Metrohm, Autolab, Riverview, FL, USA) in the Fusayama artificial saliva solution (pH 6.8 [18]) over seven days. The uncoated and coated implant samples served as the working electrodes with an area of 0.98 cm^2^ exposed to the electrolyte. The Ag|AgCl, 3.0 mol dm^−3^ KCl (*E* = 0.210 V vs. standard hydrogen electrode, SHE) was used as reference and the platinum sheet as a counter electrode. The measurements were performed using electrochemical impedance spectroscopy (EIS) at the open circuit potential (*E*_OCP_) in the frequency range from 10^4^ to 10^−3^ Hz with an ac amplitude of ±5 mV. The Solartron 1287 potentiostat/galvanostat with the FRA 1260 (Solartron Analytical, Farnborough, UK) controlled by the ZPlot^®^ software (Southern Pines, NC, USA) was used for data acquisition. The ZView^®^ software (Southern Pines, NC, USA) [19] was used for experimental data processing (χ^2^ values < 5 × 10^–3^).

### 2.4. Quantum Chemical Calculations

All the calculations were conducted at the DFT level in the Gaussian 09 (revision D.01) package [20]. Geometry optimisation was performed by the M06 functional developed by Truhlar’s group [21,22,23] and the 6-31+G(d,p) + LANL2DZ basis set. Pople’s 6-31+G(d,p) double-ξ basis set was chosen for the H, C, O, N, and P atoms, and the LANL2DZ basis set for the transition metal (Ti) atoms [24]. All the calculated structures were verified to be true minima on the potential energy surface at the same level of theory by the vibrational frequency analysis performed utilising the harmonic oscillator approximation. The thermal correction to the Gibbs free energy was derived from the same vibrational analysis. The energies were refined according to a highly flexible basis set for H, C, O, N, and P atoms, while the same LANL2DZ ECP type basis set was employed for titanium atoms. The polarizable continuum solvation model SMD, a solvation model based on density [25], was employed to account for the solvation effects. The value of a dielectric constant, ε = 78.3553 was taken for the simulation with water as solvent. The topological analysis of the charge density distribution applying Bader’s quantum theory of atoms in molecules (QTAIM) [26] was performed with the AIMALL [27] program package and utilizing the SMD/M06/6-31+G(d,p) + LANL2DZ wave function obtained from the optimisation.

All possible molecular surface/coating interactions were simulated by the (TiO_2_)_10_ nanocluster [28,29], whereas hydrolysed collagen was modelled by the functional glycine-proline-hydroxyproline tripeptide fragment, NH^3+^-Gly-Pro-Hyp-COO- [30], appearing in numerous extracellular matrix proteins as the most frequent collagen’s tripeptide unit. The Gibbs free energy of the interactions, ∆G*_INT_, was calculated using the supramolecular approach according to the formula ∆G*_INT_ = G*_AB_ − G*_A_ − G*_B_, where G*_AB_ is the total free energy of the resulting AB structure, and G*_A_ and G*_B_ are the total free energies of the associating units A and B, respectively (Tables S1 and S2 in the Supplementary Materials). A detailed description of the computational modelling is provided in the Supplementary Materials.

## 3. Results

### 3.1. The As-Received Implant—Morphological, Chemical, and Phase Analysis

**Figure 1:**
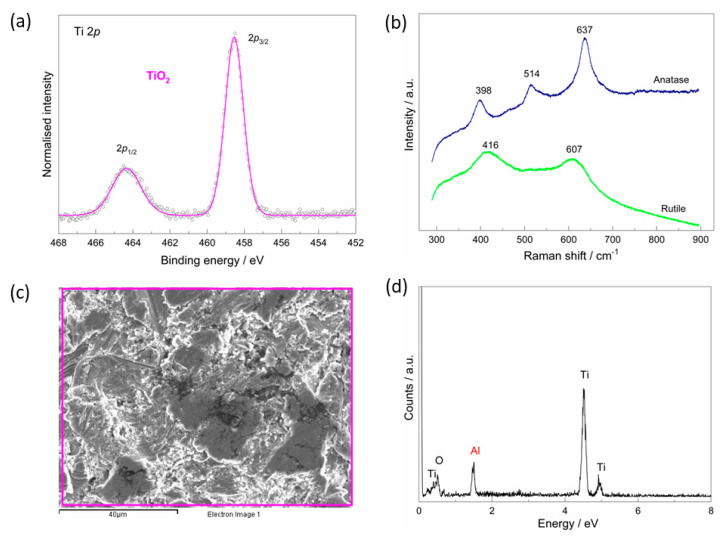
The characterisation of the as-received implant surface: (**a**) high-resolution XPS spectrum around the Ti 2*p* core level [13]; (**b**) Raman spectra of the crystalline forms of TiO_2_: rutile (green) and anatase (blue); (**c**) SEM image of the implant surface; (**d**) corresponding EDS spectrum obtained on the surface area shown in (**c**).

Chemical and phase composition and morphological features of the as-received implant were explored by SEM, EDS, Raman, and XPS techniques (Figure 1). The XPS spectrum around the Ti *2p* core level (Figure 1a) confirms the presence of the TiO_2_ on the implant’s surface, which is evident from the spin-orbit doublet characterised by the 5.8 eV difference between Ti 2*p*_3/2_ (at the binding energy, BE of 458.5 eV) and Ti 2*p*_1/2_ [31,32].

The Raman spectroscopy reveals the presence of peaks corresponding to the anatase and rutile phases of the TiO_2_ (Figure 1b) that formed during the manufacturing process. The peaks at 398 (B_1*g*_), 514 (B_1*g*_), and 637 cm^−1^ (*E_g_*) can be assigned to the anatase phase, while the peaks at 416 (*E_g_*) and 607 (A_1*g*_) cm^−1^ correspond to the rutile phase [33,34]. In the case of the rutile phase, a shift of the *E_g_* band is observed, which could be related to defects, crystallite size, or lattice strain [35,36].

The SEM shows that the TiO_2_ is inhomogeneous and microrough layer (Figure 1c) with detected aluminium probably remaining on the surface after the production process (Figure 1d).

### 3.2. The Chemical Characterisation of the Implant

**Figure 2:**
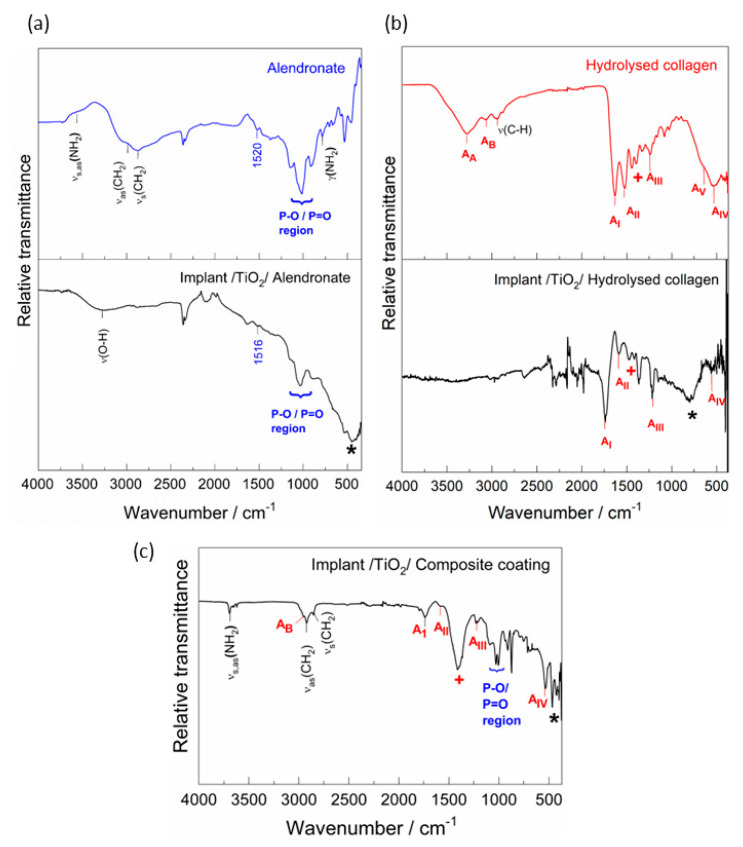
The ATR-FTIR spectra of (**a**) alendronate sodium and the implant functionalised with alendronate, (**b**) hydrolysed collagen and the implant functionalised with hydrolysed collagen, and (**c**) the implant functionalised with composite coating of alendronate and hydrolysed collagen. *: TiO_2_ vibration; +: CH_2_ wagging vibrations of glycine and proline.

An initial evaluation of the coating’s formation on the titanium implant surfaces was performed by ATR-FTIR spectroscopy (Figure 2). For comparison, the spectra of the starting chemicals, alendronate sodium and hydrolysed collagen, are shown. In addition, all spectra are compared with the vibrational spectra calculated by DFT (Supplementary Materials Figure S1).

The IR spectra of the alendronate and the implant/alendronate samples (Figure 2a) exhibit a characteristic P−O and P=O region (~1200–900 cm^−1^), and the phosphate band at 1520 (1516) cm^−1^ characteristic for the alendronate [37,38]. The bands are slightly altered by the interaction between alendronate and implant’s surface. The effect of alendronate adsorption is most pronounced in the high wavenumber region resulting in the absence of the NH_2_ band (around 3580 cm^−1^). A wide band in the range between ~800 and 400 cm^−1^ (marked with *) can be assigned to the TiO_2_ layer vibration according to the DFT spectrum (Figure S1). This band appears in the spectra of all implant samples. The result is in accordance with the results of XPS and Raman that confirmed the existence of the TiO_2_ on the implant surface (Figure 1a,b).

The spectrum of the hydrolysed collagen (Figure 2b) shows characteristic bands of peptide binding vibrations: amide I (stretching vibration of −C=O of amide group at 1631 cm^−1^), amide II (N−H stretching coupled to the C−N stretching of amide group at 1522 cm^−1^), and amide III (C−N stretching and N−H in-plane bending of amide linkage at 1240 cm^−1^) [39,40,41]. The vibrations of amide IV (ν(C−C) and δ(O−C−N) at 540 cm^−1^) and amide V (δ(N−H) at 670 cm^−1^) are also visible [40,41]. The presence of water molecules forming hydrogen bonds with collagen molecules is reflected in the bands of amide A (ν(−OH) at 3280 cm^−1^) and amide B (ν(N−H stretching) at 3067 cm^−1^) [40]. The bands in the range 1400–1200 cm^−1^ (marked with +) can be assigned to the CH_2_ wagging vibrations of glycine and proline [39]. The functionalisation of the implant by the hydrolysed collagen resulted in the disappearance of some bands, the change in the intensity of the bands, and the shift of the peaks to higher wavenumbers, as can be seen in Figure 2b. Hydrogen bonds and conformational changes (confirmed by DFT, Section 3.3), which are characteristic of the collagen molecule, influence the shift of the bands. All this indicates successful adsorption of the hydrolysed collagen molecules on the implant’s surface.

The bands of the collagen peptide bond and the P−O and P=O bonds characteristic for the alendronate are visible in the spectrum of the implant/composite coating, Figure 2c. All spectra confirm that the coatings have successfully formed on the implant surfaces.

**Figure 3:**
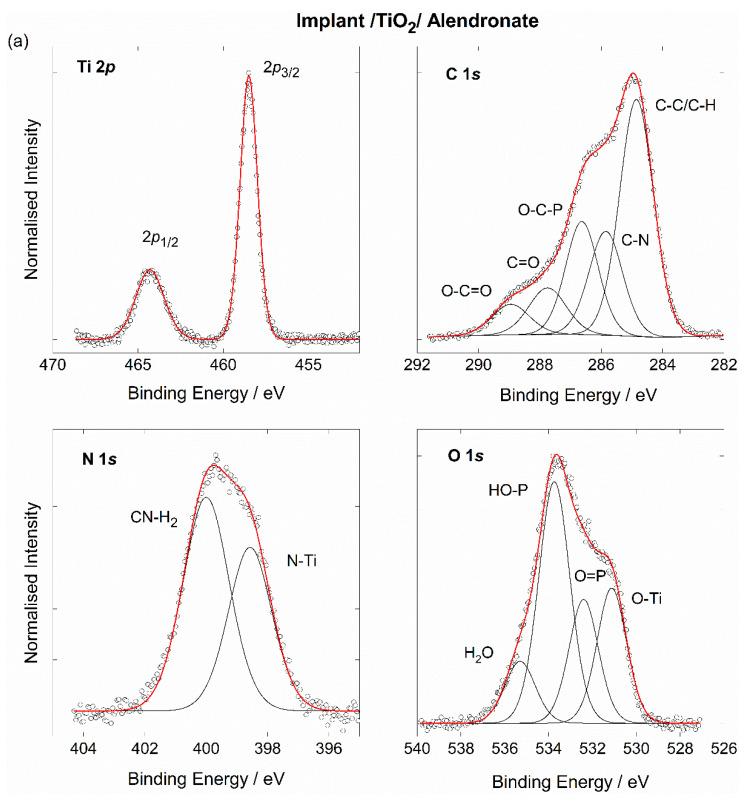

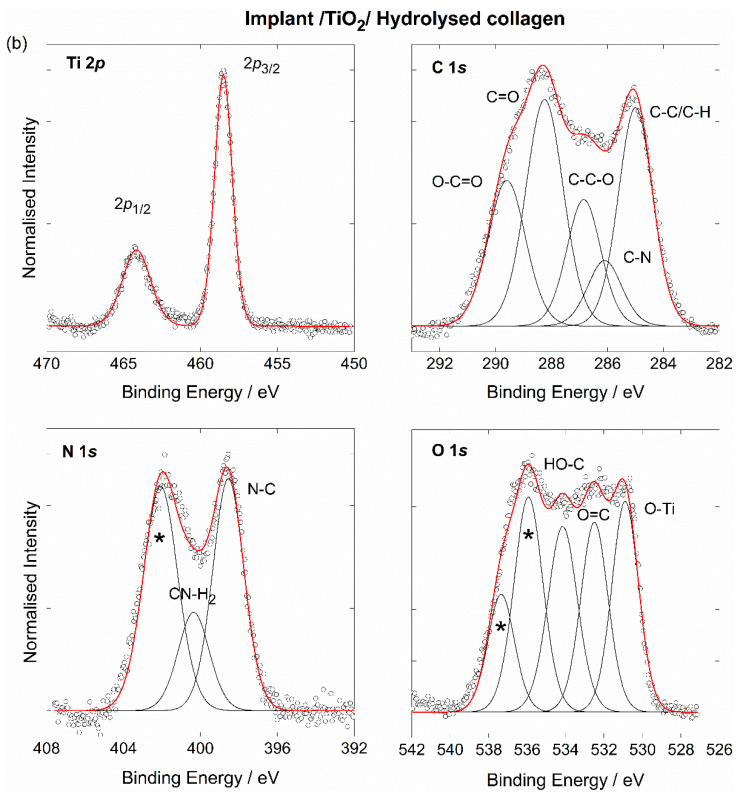

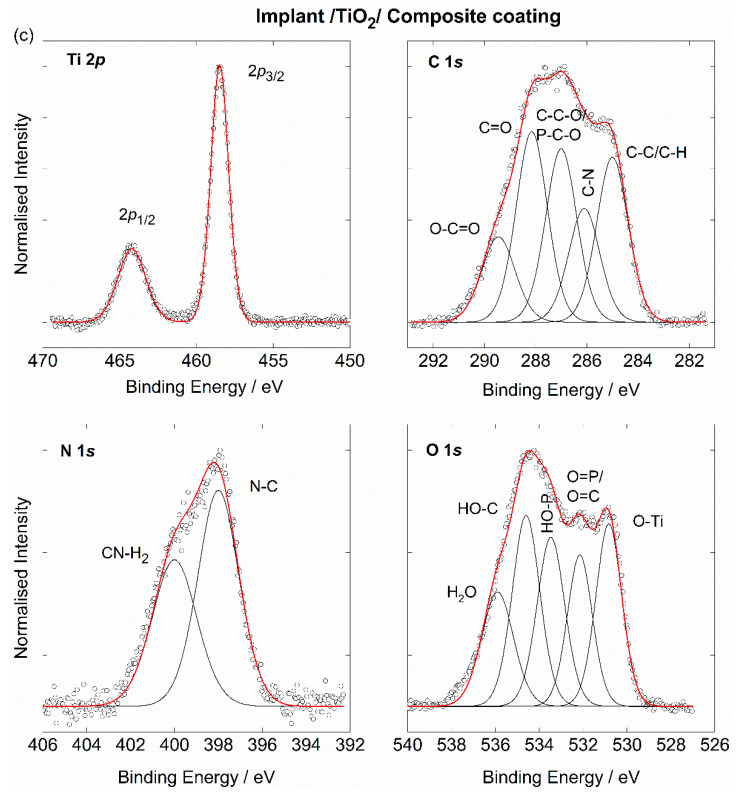
High-resolution XPS spectra around Ti 2*p*, C 1*s*; N 1*s*, O 1*s* core levels of (**a**) the implant functionalised with alendronate [13], (**b**) the implant functionalised with hydrolysed collagen, and (**c**) the implant functionalised with composite coating. Symbols: experimental data; black lines: main contributions; red line: total fit; *: contamination.

The XPS spectra around the Ti 2*p* core levels for all samples examined (Figure 3) reveal a structure of Ti atoms in the TiO_2_ compound characterised by a well-separated Ti 2*p*_3/2_ and Ti 2*p*_1/2_ spin-orbit doublet with the Ti 2*p*_3/2_ component at BE of 458.5 eV and the energy separation of 5.8 eV between the two peaks [31]. The result is in good agreement with the Raman spectra of the as-received implant (Figure 1b), which confirm the presence of TiO_2_ on the implant’s surface.

On the other hand, the different structure of the XPS peaks found in different samples around C 1*s*, O 1*s*, and N 1*s* core levels reflects the differences in atomic chemical bonding of the organic coatings studied in the present work (Figure 3). Thus, as assigned in our previous work [13], the three main contributions found in the C 1*s* spectrum of the alendronate-coated implant, are related to the C–C (285.0 eV), C–N (286.0 eV), and P–C–O (286.6 eV) bonds [42], while the low-intensity peaks at the higher BE side of the C 1*s* curve are attributed to surface oxygen contamination (O–C=O, C=O; see Figure 3a). In contrast, the C 1*s* spectra of the implants coated with the hydrolysed collagen and the composite coating (Figure 3b,c) show a quite different structure, with the intense peaks at BEs of 288.2 eV and 289.5 eV attributed to C=O and O–C=O bonds, respectively [43,44].

The deconvoluted N 1*s* spectrum of the alendronate-modified implant shows the two distinguished components, related to the nitrogen atoms in the C–NH_2_ bond (400.0 eV) and the N atoms bonded to the Ti atoms of the implant (398.5 eV), as shown in Figure 3a [12,13]. While the C–NH_2_ component is present in the N 1*s* spectra of the implants coated with the hydrolysed collagen and the composite coatings, no contribution from nitrogen bonded to the implant was observed in these two samples. This strongly suggests that the –NH_2_ group remained free and unbounded to the TiO_2_-covered implant and, therefore, can influence the surface properties of the implants modified with hydrolysed collagen and composite coatings. The peak at BE of 398.0 eV can be related to the N–C bond in the collagen molecule [43,44]. The intense peak (marked with *) at the higher BE side of the N 1*s* peak of the sample modified with composite coating can be attributed to surface contamination in the form of oxidised nitrogen species [45].

Turning now to the photoemission spectra around O 1*s* atomic levels in alendronate and collagen molecules (Figure 3a,b), the three characteristic fitting components were assigned to oxygen bonded in O=P/O=C (532.4 eV), HO–P (533.7 eV) and HO–C (534.3 eV) configuration [13,46,47], in addition to the O atoms bonded to titanium (peak at BE of 531.0 eV) [13,46]. Some additional contributions are visible in the O 1*s* spectra (marked with *), reflecting the presence of surface species, most likely in the form of adsorbed water molecules [48] or fluorine contamination [49].

### 3.3. The Coating’s Formation Mechanism on the Implant

To understand the coating’s formation mechanism between the TiO_2_-covered implant and the selected coating molecules, a detailed theoretical study using quantum chemical calculations at the density functional theory (DFT) level was performed. The small (TiO_2_)_10_ nanocluster was used for cluster modelling of the titanium surface [29], while hydrolysed collagen was modelled by the functional glycine-proline-hydroxyproline tripeptide fragment, NH^3+^-Gly-Pro-Hyp-COO^−^ [30].

**Figure 4:**
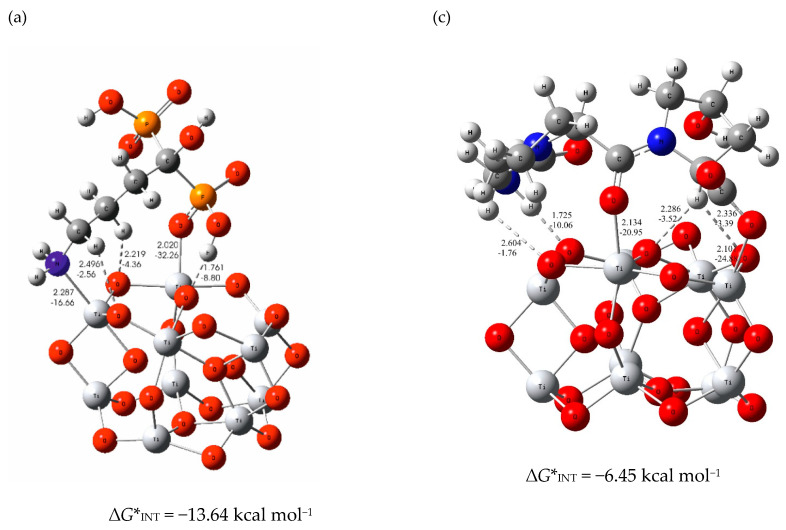

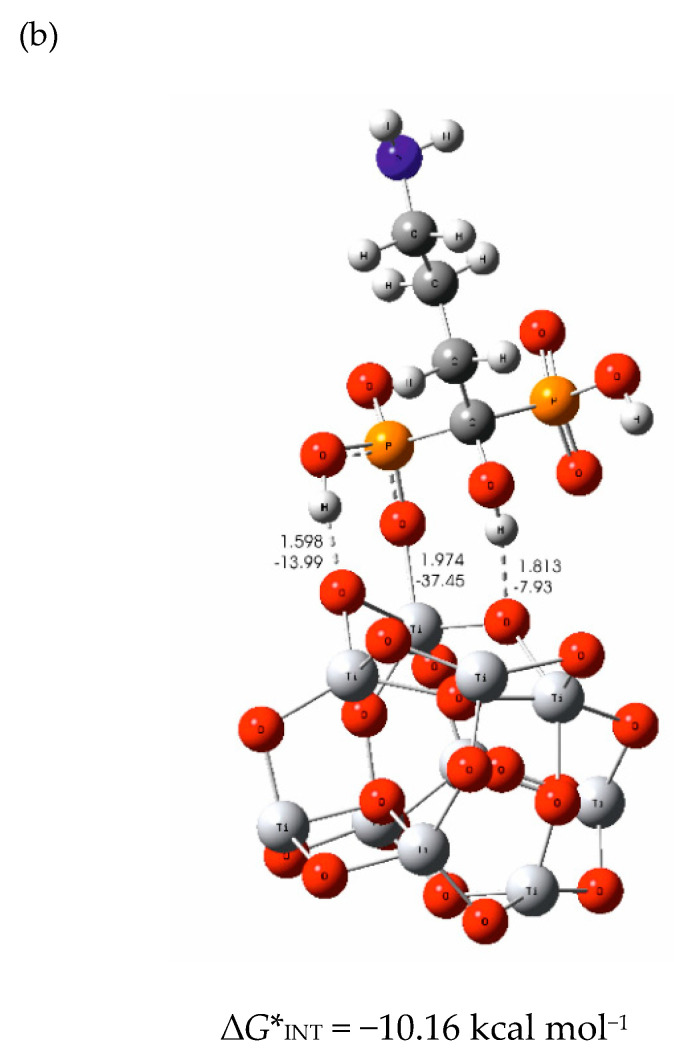
The most stable structures predicted by DFT of (**a**,**b**) the (TiO_2_)_10_─alendronate [13] and (**c**) the (TiO_2_)_10_─tripeptide representing hydrolysed collagen. Bond distances are in Å and bond energies are in kcal mol^−1^. Oxygen—red ball; Nitrogen—blue ball; Phosphorus—orange ball; Titanium—light grey ball.

The large difference in the values of Gibbs free energies obtained for the most stable (TiO_2_)_10_—alendronate (∆G*_INT_ = −13.64 kcal mol^−1^; Figure 4a,b) and (TiO_2_)_10_—tripeptide (∆G*_INT_ = −6.45 kcal mol^−1^, Figure 4c) molecular interactions suggests a more spontaneous formation of the alendronate coating on the titanium implant. The most stable (TiO_2_)_10_—tripeptide structure is the result of two coordinate Ti–O bonds (C–O–Ti) additionally accompanied by hydrogen bonds (Figure 4c). The formation of the (TiO_2_)_10_—alendronate coating is most likely the result of two energetically competitive structures, one in which the alendronate molecule is bound to the surface via both the amine (–NH_2_) and phosphonate (–PO_3_H) groups (∆G*_INT_ = −13.64 kcal mol^−1^), and the other in which the alendronate molecule is bound via the phosphonate (–PO_3_H) group (∆G*_INT_ = −10.16 kcal mol^−1^; Figure 4a,b) [13]. All the structures described above are additionally stabilised by hydrogen bonds.

**Figure 5:**
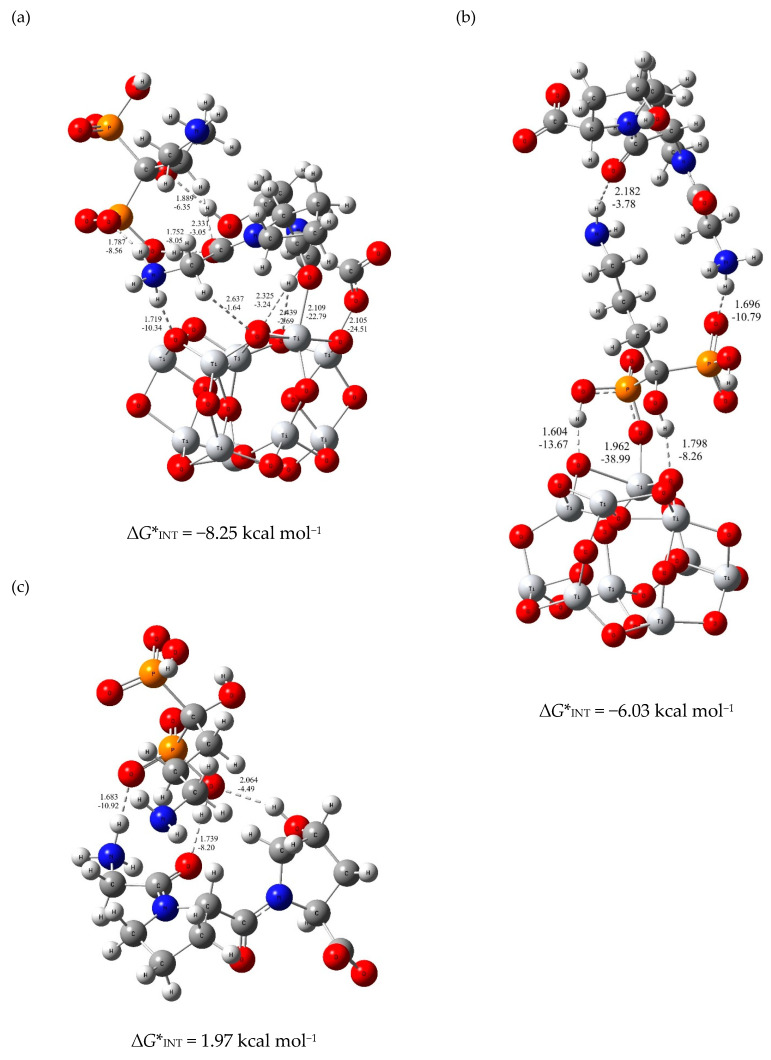
The most stable structures, predicted by DFT for (**a**) (TiO_2_)_10_─alendronate─tripeptide, (**b**) (TiO_2_)_10_─alendronate─tripeptide, and (**c**) starting tripeptide─alendronate structure. Bond distances in Å and bond energies in kcal mol^−1^. Oxygen—red ball; Nitrogen—blue ball; Phosphorus—orange ball; Titanium—light grey ball.

Two different strategies were used to model molecular interactions between the TiO_2_ layer on the implant and the composite coating molecules. One of the strategies is to model the composite component molecules simultaneously, and the other is to gradually add component molecules during the interaction simulation calculation. The results of the second approach, provided in the Supplementary Materials (Section B), yielded in less stable configurations (higher ∆G*_INT_ values). In the case when tripeptide and alendronate molecules are taken into account simultaneously during the DFT calculation, the formation of the composite coating occurs most likely through two energetically competitive structures. In one the tripeptide unit of the collagen is bound to the TiO_2_ layer as the inner part of the coating, while alendronate is oriented toward the outer part of the coating ((TiO_2_)_10_—tripeptide—alendronate, ∆G*_INT_ = −8.25 kcal mol^−1^; Figure 5a). The bonding between tripeptide and TiO_2_ surface occurs via two strong coordinate (C–O–Ti) bonds of the amino acid branches (d_Ti–O_ value up to 2.105 Å, *E*_Ti–O_ value up to −24.51 kcal mol^−1^) supported by one N–H∙∙∙O (d_O∙∙∙H_ = 1.719 Å, *E*_O∙∙∙H_ = −10.34 kcal mol^−1^) and three C–H∙∙∙O hydrogen bonds (d_O∙∙∙H_ value up to 2.325 Å, *E*_O∙∙∙H_ value up to −3.24 kcal mol^−1^). In the other structure, the alendronate molecule is bound to the titanium surface via phosphonate group (–PO_3_H) as the coating’s inner part with tripeptide as the coating’s outer part ((TiO_2_)_10_–alendronate─tripeptide; ∆G*_INT_ = −6.03 kcal mol^−1^; Figure 5b). The bonding occurs via coordinate (P–O–Ti) bond, (d_Ti–O_= 1.962Å, *E*_Ti–O_ = −38.99 kcal mol^−1^) supported by two O–H∙∙∙O hydrogen bonds, Figure 5b. The coordinate Ti–O bonds are attributed to an ionic type of interaction according to ∇^2^*ρ(r*_c_*)* > 0 and *H(r*_c_*)* > 0.

It is important to point out that weak intermolecular interactions between alendronate and tripeptide occur immediately at the beginning of the coating process forming an initiating linker or “coating directing agent”, Figure 5c. Due to a lower flexibility influenced by the presence of three hydrogen bonds O–H∙∙∙O, N–H∙∙∙O, and C–H∙∙∙O, alendronate molecules as part of the coating can only participate in the interactions with the phosphonate group (Ti–O), as shown in Figure 5b. For this reason, to model the (TiO_2_)_10_—alendronate—tripeptide structure, the less stable (TiO_2_)_10_—alendronate structure (Figure 4b) is used. Most likely, the composite coating formation is a result of both structures (Figure 5a,b) that would compete energetically and provide pronounced dynamics of a formation process.

### 3.4. The Electrochemical Behaviour of Implants in Artificial Saliva Solution

**Figure 6:**
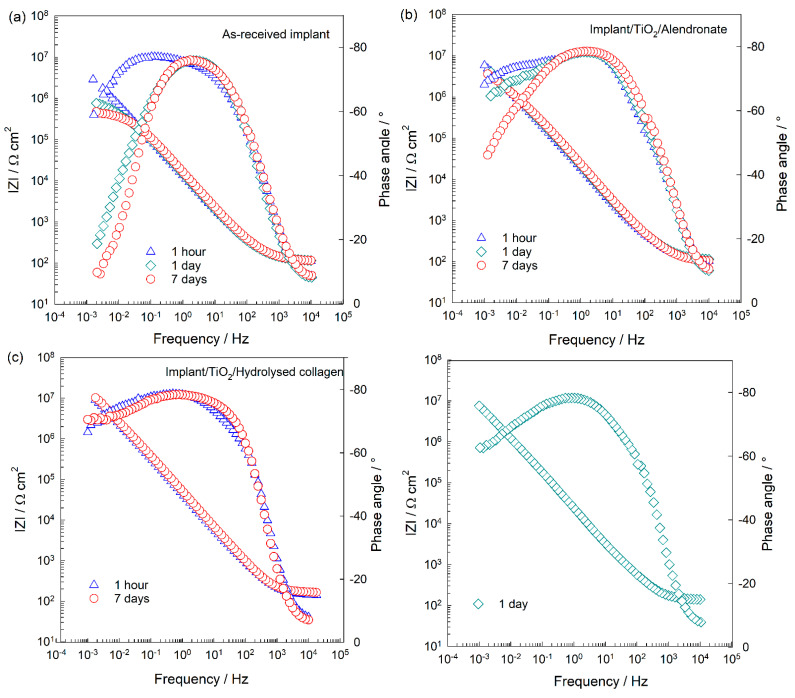

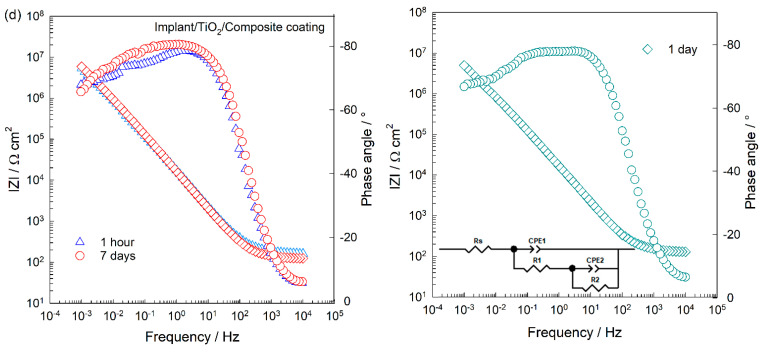
Bode plots of (**a**) as-received implant [13], (**b**) implant/TiO_2_/alendronate coating [13], (**c**) implant/TiO_2_/hydrolysed collagen coating, and (**d**) implant/TiO_2_/composite coating recorded after a stabilisation time of 1 h, 1 day, and 7 days at the open circuit potential in the artificial saliva solution, pH = 6.8. The insert: EEC used to model data.

The electrochemical investigations of the implants were carried out at the open circuit potential (*E*_OCP_) over 7 days of immersion in artificial saliva solution (1 h to 7 days). The results are shown in the form of the Bode plots (Figure 6), while the Nyquist plots are shown in the Supplementary Materials (Figure S3).

The structure of the electrified implant/artificial saliva interface can be described by an electrical equivalent circuit (EEC) with two time constants that are characteristic for a two-layer oxide film, TiO_2_ (inset in Figure 6d) [48,49]. Modelling results are given in Table 1. Due to the microscopic inhomogeneities of the studied system, a constant phase element (CPE) was used instead of a capacitor (C) [50,51]. The interfacial capacitance (*C*) values were calculated using Brug’s equation [51]. *R*_s_ is the electrolyte resistance.

The high-frequency time constant (*R*_1_CPE_1_) is related to the resistance (*R*_1_) and capacitance (CPE_1_) of the outer porous part of the oxide film, while the low-frequency time constant (*R*_2_CPE_2_) is related to the resistance (*R*_2_) and capacitance (CPE_2_) of the inner barrier part of the oxide. After 1 h of immersion in the artificial saliva (Figure 6a), the as-received implant possesses good protective properties, which can be attributed to the inner part of the TiO_2_ (*R*_2_ is higher than *R*_1_). Gradually, the protective properties of the oxide deteriorated and after 7 days of immersion, the *R*_2_ value decreased by ~23 times. Since *R*_2_ values are related to pores of the outer part of the oxide, obviously the density and/or size of pores increases with time allowing for ion/water diffusion from the solution deeper into the oxide. Consequently, *R*_2_ values decrease with time.

To improve the chemical stability of the implants, alendronate, hydrolysed collagen, and composite coatings were formed as additional barriers on the TiO_2_-covered implant surfaces, and their EIS spectra are presented in Figure 6b–d. A brief inspection of the EIS responses shows that all coatings have a positive effect on the protective properties of the implants (higher values of log |*Z*| versus log *f* compared to the values of the as-received implant; Figure 6), but their structural properties are different. The implant/TiO_2_/coating/saliva interface is described by the same EEC with two time constants, and the modelled values are listed in Table 1. The high/middle-frequency time constant (*R*_1_CPE_1_) is related to the resistance and capacitance of the surface film (organic coating over TiO_2_), while the low-frequency time constant (*R*_2_CPE_2_) is related to the resistance and capacitance of film structural defects [52]. The polarisation resistance, *R*_p_ [53] as a direct measure of material’s corrosion protection, is the sum of the values of *R*_1_ and *R*_2_ and allows for a calculation of the anti-corrosion effectiveness of the coated implant samples, *η*; *η* = (*R*_p,modified_ − *R*_p,unmodified_)/*R*_p,modified_. *R*_p,modified_ is the polarisation resistance of the coated implant and *R*_p,unmodified_ of the as-received implant.

In the case of the alendronate-modified implant, its protective properties and stability slightly decrease over time. The alendronate coating contains structural imperfections, pores that are visible from the phase angle (*θ* ˂ 90°), and n_2_ values (n_2_ ˂ 1). Decrease of the n_2_ values with time points to the propagation, additional occurrence of pores, and/or desorption of the molecules, which is induced by the hydrophilic character of the alendronate coating [13]. Structural defects influence negatively on the resistivity of the implant. Therefore, the *R*_2_ decrease is connected directly with the coating’s defects. The alendronate coating provides 92% protection to the implant during the 7-day immersion to the saliva.

The coating of the hydrolysed collagen provides a high protection of 99% to the implant during 7-day immersion in the saliva (Figure 6c). However, after 1 day of immersion, a sharp decrease of the *R*_2_ value occurs. Obviously, a contact of the coating´s unbonded functional groups with the ions of the electrolyte initiates a restructuring and the compactness of the coating is impaired. Once the peptide chains are reassembled into the stable and compact structure (DFT, Figure 4c), the *R*_2_ value increases again, Table 1.

The EIS responses of the implant functionalised with the composite coating remain almost unchanged during the 7-day immersion to the artificial saliva (Figure 6d). The interaction of the coating with the saliva is pronounced in the first day of the immersion when the coating reaches a less compact structure (reflected in *θ* versus log *f*). Consequently, *R*_2_ values decrease. Inter- and intramolecular interactions present in the composite coating (Figure 5a,b) enable a fast reorganisation of molecules resulting in the compact and stable structure (n_2_ and *R*_2_ values increase slightly).

## 4. Discussion

Although the implant used in this study was covered with the protective TiO_2_ layer (confirmed by Raman and XPS; Figure 1b,c), its protective properties deteriorated during a short period of 7-day immersion in the artificial saliva (Figure 6a). Since an aggressive environment such as saliva can induce a degradation of the implant, dissolution, and release of metal ions in the surrounding organs, it was attempted to improve the protective properties of the implant by means of the surface coatings formation.

The DFT calculations (Figure 4 and Figure 5) showed that the formation of all types of coatings investigated is spontaneous and ATR-FTIR (Figure 2) and XPS (Figure 3) confirmed their existence on the TiO_2_-covered implant surface.

The correlation of the DFT and XPS results enabled the determination of all possible molecular interactions and binding pathways of molecules to the implant’s surface. The knowledge of inter- and intramolecular interactions between coating molecules and the implant was essential for the understandings of the electrochemical behaviour of modified implants in the artificial saliva (Figure 6). Although all prepared coatings clearly showed a positive influence on the stability and resistivity of the implant in comparison to the unmodified implant, EIS results revealed structural finesses of the coatings responsible for the electrochemical stability of the modified implants.

The alendronate coating showed the most decreased protection in the artificial saliva (Figure 6b) among all coatings, although the interactions between implant and alendronate molecules are the most stable according to the DFT (Figure 4 and Figure 5). The surface of the implant modified with the alendronate is hydrophilic due to the presence of free hydrophilic functional groups –NH_2_, –COH, and –PO_3_H in the outer part of the coating (Figure 4a,b). It is well known that the hydrophilicity of the material has a negative effect on corrosion protection due to possible interactions with molecules/ions from electrolytes [13,52]. These interactions can cause a structural reorganisation of the coating, its desorption, and/or occurrence of defects that enable a penetration of ions from electrolytes to the underlying implant. As a result, corrosion protection and stability of the implant are reduced.

A high protection efficiency of the implant in the artificial saliva was achieved by the coating of the hydrolysed collagen (Figure 6c). The hydrolysed collagen as a biopolymer tends to form a crosslinking network, which includes various non-covalent, hydrophobic, and ionic interactions, as well as hydrogen and coordination bonds (DFT, Figure 4c). Furthermore, peptide chains of the hydrolysed collagen are broken into smaller parts that tend to organise into fibrils and are further stabilised through hydrogen bonding and crosslinking [14,54] All the interactions result in a compact structure of the coating and maintain its stability during the exposure of the implant to the saliva solution (Figure 6c).

The composite coating is most likely bound complexly to the implant via two stable structures (Figure 5a,b), which behave differently in contact with the saliva solution. If alendronate molecules are at the outer interface implant/coating/saliva (Figure 5a), restructuring of the coating can occur due to the interactions between the free hydrophilic groups (–NH_2_ and –PO_3_H) of the alendronate and the water/ions of the saliva. There is also a possibility that the alendronate molecules are desorbed from the implant. Consequently, the collagen layer of the coating will restructure. If collagen molecules are at the outer interface implant/coating/saliva (Figure 5b), the organisation of peptide chains in fibrils and/or their crosslinking can occur. All possible interactions reflect positively on the stability and protection of the implant in contact with the artificial saliva (Figure 6d).

From an application point of view, the composite coating of alendronate and hydrolysed collagen would be optimal for the functionalisation of the implant. Its high corrosion protection and stability are basic prerequisites for a successful long life of implants in the oral cavity. The combination of alendronate, a strong osteoinductive molecule, and collagen, a biopolymer that provides structural and mechanical support to bone and connective tissue, could induce and accelerate the osseointegration of the implant in the human body. The influence of the implant prepared in this way on long-term corrosion protection and the process of osseointegration needs to be investigated in the future.

## 5. Conclusions

The coatings of alendronate sodium and hydrolysed collagen, as well as the composite coating, were formed successfully by self-assembly process on the titanium dental implants.

The Raman and XPS analyses reveal the presence of TiO_2_ in the form of rutile and anatase phase on the implant’s surface.

In addition, the DFT results show that the molecular interactions between the TiO_2_-covered implant surface and the organic molecules were spontaneous (∆G*_INT_ < 0). The value of the Gibbs free energy is the most stable for (TiO_2_)_10_─alendronate ∆G*_INT_ = −13.64 kcal mol^−1^, for (TiO_2_)_10_─tripeptide ∆G*_INT_ = −6.45 kcal mol^−1^, and for (TiO_2_)_10_─tripeptide─alendronate ∆G*_INT_ = −8.25 kcal mol^−1^.

Furthermore, the structure of the alendronate coating is the result of two energetically competitive structures. The bonding occurs via the amine (–NH_2_) and the phosphonate (–PO_3_H) groups (∆G*_INT_ = −13.64 kcal mol^−1^), and/or only via the phosphonate (–PO_3_H) group (∆G*_INT_ = −10.16 kcal mol^−1^). Hydrophilic (–NH_2_), (–COH), and (–PO_3_H) groups in the outer part of the coating negatively influence the electrochemical stability of the modified implant, whereas the protection efficiency decreases with time and reaches 92% after 7-day immersion in the artificial saliva.

The hydrolysed collagen coating is bound to the implant via two coordinate Ti–O bonds (C–O–Ti) stabilised by hydrogen bonds. Additionally, the crosslinking between peptide chains has a key role for the stability of the modified implant. The modified implant is protected with high protection efficiency of 99% after the 7-day immersion in the artificial saliva.

The final structure of the composite coating is a result of the competition between two stable structures (TiO_2_)_10_─tripeptide─alendronate (∆G* _INT_ = −8.25 kcal mol^−1^) and (TiO_2_)_10_─alendronate─tripeptide (∆G* _INT_ = −6.03 kcal mol^−1^). Hydrogen bonds and crosslinking of the peptide chains, characteristic for both structures, contribute to the high protection efficiency of 98% after the 7-day immersion in the artificial saliva.

## Figures and Tables

**Table 1:** Impedance parameters calculated from EIS data (Figure 6) for the as-received implant and functionalised implant samples.

Samples	*R*_s_/Ω cm^2^	CPE_1_∙10^6^/ Ω^−1^ cm^−2^ s^n1^	n_1_	*C*_1_/µF cm^−2^	*R*_1_/Ω cm^2^	CPE_2_∙10^6^/ Ω^−1^ cm^−2^ s^n1^	n_2_	*C*_2_/µF cm^−2^	*R*_2_/MΩ cm^2^	*η/*%
AS-RECEIVED IMPLANT										
1 h	111	9.98	0.853	3.02	760	5.16	0.850	1.38	9.90	
1 day	123	2.88	0.997	2.88	174	12.1	0.788	2.10	0.79	
7 days	123	3.26	0.978	2.71	307	9.21	0.810	1.88	0.44	
IMPLANT/TiO_2_/ALENDRONATE										
1 h	109	2.00	1	2.00	307	9.21	0.820	2.03	39.0.	74.6
1 day	119	1.95	1	1.95	332	8.05	0.806	1.48	16.5	95.2
7 days	109	1.98	1	1.98	302	7.23	0.795	1.15	5.88	92.5
IMPLANT/TiO_2_/HYDROLYSED COLLAGEN										
1 h	149	2.03	1	2.03	851	5.46	0.815	1.07	54.5	81.8
1 day	147	2.09	1	2.09	641	6.45	0.809	0.91	24.0	96.7
7 days	160	1.95	1	1.95	501	5.40	0.782	0.45	60.1	99.3
IMPLANT/TiO_2_/COMPOSITE COATING										
1 h	169	3.22	1	3.22	182	9.01	0.804	1.14	29.4	66.3
1 day	137	3.30	1	3.30	176	9.25	0.801	0.99	23.7	96.0
7 days	130	3.85	1	3.85	205	8.33	0.826	1.26	24.0	98.2

## Data Availability

The data presented in this study are available on request from the corresponding author.

## Supplementary Materials

The following supporting information can be downloaded at: https://www.mdpi.com/article/10.3390/ma15155127/s1, Figure S1: Theoretically calculated IR spectra of the investigated systems, Figure S2: Optimised structures of the selected systems (bond distances in Å, bond energies in kcal mol^−1^), Table S1: Formation of single-layer and two-layer coatings. Standard state (1M) free energies of interaction Δ_r_*G**_INT_ computed by using the SMD solvation model at the M06/6-311++G(2df,2pd) + LANL2DZ// M06/6-31+G(d,p) + LANL2DZ level of theory (in kcal mol^−1^), Table S2: Total electronic energy, E^Tot^_soln_, obtained at the SMD/M06/6-311++G(2df,2pd) + LANL2DZ//SMD/M06/6-31+G(d,p) + LANL2DZ level of theory; thermal correction to the Gibbs free energy, Δ*G**_VRT,soln_, obtained at the SMD/M06/6-31+G(d,p) + LANL2DZ level of theory; and total free energy, *G**_X_, (*G**_X_ = E_Totsoln_ + Δ*G**_VRT,soln_) in water media of the investigated species (all energies in hartree), Table S3: Bond lengths (d), energies (E), and QTAIM properties of the selected bonds in the investigated systems; Cartesian coordinates of the calculated systems, Figure S3: Nyquist plots of (a) as-received implant, (b) implant/TiO_2_/alendronate coating, (c) implant/TiO_2_/hydrolysed collagen coating, and (d) implant/TiO_2_/composite coating recorded after a stabilisation time of 1 h, 1 day, and 7 days at the open circuit potential in the artificial saliva solution, pH = 6.8 [55,56,57,58,59,60,61,62,63,64,65].
