# Supplementary material for: Surface Functionalisation of Dental Implants with a Composite Coating of Alendronate and Hydrolysed Collagen: DFT and EIS Studies
Source: Materials (Basel). 2022 Jul 23;15(15):5127. doi: 10.3390/ma15155127 (PMC9351680; doi:10.3390/ma15155127)
Supplement: Supplementary file 1 [file materials-15-05127-s001.zip › materials-1768324-SM.pdf]

Supplementary Materials

A) Computational modeling. All calculations were performed by means of quantum chemical calculations at the density functional theory (DFT) level using the Gaussian 09 program (revision D.01) [20]. The $(TiO_2)_n$ clusters were employed, proposed by Allard et al. [28] and Qu and Kroes [29] with n = 10.

The M06 functional designed by the Truhlar's group, which provides very accurate thermodynamic parameters, being particularly successful in nonbonding interactions treatment, has been selected [21-23]. The 6-31+G(d,p) + LANL2DZ mixed basis set has been utilized. The Pople's 6-31+G(d,p) double-ξ basis set was chosen for O, H, C, N, P atoms and the LANL2DZ basis (LANL2 pseudopotential for inner electrons and its associated double-ξ basis set (DZ)) was used for the transition-metal (Ti) atoms [24]. This gave rise to the M06/6-31+G(d,p) + LANL2DZ model utilized for geometry optimization which has been frequently used for studies of transition-metal containing systems. The geometric structures of the molecules were optimized by minimizing energies with respect to all geometrical parameters without imposing any molecular symmetry constraints and using a tight convergence condition. The Berny algorithm using redundant internal coordinates was employed. Frequency calculations were made under the harmonic approximation on all the optimized structures at the same level of theory with no scaling in order to confirm that the structures correspond to the true minima, meaning that no imaginary frequencies were present, as well as to extract thermal Gibbs free energy corrections. The final single point energies were obtained using a highly flexible 6-311++G(2df,2pd) basis set for the O, H, C, N, P atoms, while the same LANL2DZ ECP type basis set for titanium atoms was employed.

The self- consistent field (SCF) calculations were conducted under a tight condition imposing the threshold value of 10^{-8} hartree to total energy difference during the iteration process. The integration grid was set to FineGrid having 75 radial shells and 302 angular points per shell. The 2-electron integral accuracy was set to 10^{-13}. The FoFCou algorithm with NoSymm option was utilized. All the geometry optimizations, frequency calculations and single point energy evaluations were performed by taking solvent effects into account. To evaluate the bulk solvent effects (water, ε = 78.3553 at 25°), the implicit SMD polarizable continuum solvation model [25] has been employed. It represents a very practical approach to simulate the solvation environment and determine the effect of the medium on the structure and stability of solutes in a solution. The conformational space was manually sampled for the $(TiO_2)_{10}$—alendronate taking into account various donor and acceptor sites of the $(TiO_2)_{10}$ cluster and alendronate molecule. Two thermodynamically most stable $(TiO_2)_{10}$—alendronate structures are reported here. The starting structure of the alendronate was taken from the literature [55] and modified subsequently to a dianionic form.

The interaction Gibbs free energies, ΔG^*_{INT} were computed as the difference between the total free energy (G^*_{AB}) of the $(TiO_2)_{10}$—alendronate structure and the sum of the total free energies ($G^*_A + G^*_B$) of the associating units A and B ($(TiO_2)_{10}$ cluster and alendronate molecule) using the supramolecular approach:

$$\Delta G^*_{INT,AB} = G^*_{AB} - G^*_A - G^*_B \quad (S1)$$

The species total free energy in the liquid was calculated using the expression:

$$G^*_X = E^{Tot}_{soln} + \Delta G^*_{VRT,soln} \quad (S2)$$

where E^{Tot}_{soln} corresponds to the basic energy of a density functional theory calculation using the SMD model, while $\Delta G^*_{VRT,soln}$ encompasses vibrational, rotational and translational contribution to the solution free energy, being computed by applying the ideal gas partition functions to the frequencies calculated in the dielectric medium and the 1M standard state. A more negative value of the binding energy implied the more stable formed species. No BSSE correction of binding energies has been applied.

The topological analysis of the charge density distribution using the Bader's quantum theory of atoms in molecules (QTAIM) [26] was performed by employing AIMALL software package [27] using the

SMD/M06/6-31+G(d,p) + LANL2DZ wave function obtained from optimization. Within the QTAIM analysis the electron density was analysed for two major characteristics: (a) the existence of critical points (CPs), where electron density exhibits maximum, minimum, or a saddle point in space, and (b) for the bond paths [56], the maximum electron density line connecting two interacting atoms in the energetic minimum structure (the two atoms are bonded). The point of the electron density minimum value along that line called the bond critical point (BCP) and values of topological parameters, like electron density $\rho(r_c)$, Laplacian $\nabla^2\rho(r_c)$, electronic kinetic energy $G(r_c)$, electronic potential energy density $V(r_c)$, total energy density $H(r_c)$ at that point explain interatomic interaction features. $\nabla^2\rho(r_c) < 0$ indicates locally concentrated charge density, while locally depleted charge density is indicated by $\nabla^2\rho(r_c) > 0$. The chemical bond nature can be described qualitatively concerning signs and values of the electron density Laplacian $\nabla^2\rho(r_c)$ and the electron energy density $H(r_c)$ at the bond critical point according to following criteria. The interactions characterized by $\nabla^2\rho(r_c) < 0$ and $H(r_c) < 0$ (shared interaction) are characteristic for weakly polar and nonpolar covalent bonds. On the other hand, $\nabla^2\rho(r_c) > 0$ and $H(r_c) > 0$ (closed shell interactions) point to ionic bonds, weak hydrogen bonds, and van der Waals interactions. The intermediate interactions which includestrong hydrogen bonds and most of the coordinate bonds are characterized by $\nabla^2\rho(r_c) > 0$ and $H(r_c) < 0$ [57,58]. A very high negative value of the $\nabla^2\rho(r_c)$ is an indication of a strong covalent bond, while a high positive value corresponds to a strong noncovalent bond. The energies of the coordinate bonds (Ti—O, Ti—N) and of other intermolecular hydrogen bonds have been calculated by the Espinosa's equation [59]:

$$E = 0.5\ V(r_c) \quad \text{(S3)}$$

where E is the bond energy (a.u.), and $V(r_c)$ is potential energy density (a.u.) at the corresponding critical point. The Espinosa's relationship is widely used for the energy estimation of different types of hydrogen [60,61], van der Waals [62], coordinate [63,64], and homopolar bonds [65].

B) The composite coating's formation mechanism on the implant: – supplementary addition of molecules

When the model of supplementary addition of alendronate molecules to the isolated $(TiO_2)_{10}$—tripeptide as well as tripeptide molecules to the isolated $(TiO_2)_{10}$—alendronate is used instead of the model of simultaneous use of both molecules, the calculations show decreased stability of the system containing the species $(TiO_2)_{10}$—tripeptide—alendronate with ΔG^*_{INT} = -1.08 kcal mol^{-1} (alendronate + $(TiO_2)_{10}$—tripeptide → $(TiO_2)_{10}$—tripeptide—alendronate) as well as $(TiO_2)_{10}$—alendronate—tripeptide with ΔG^*_{INT} = 4.13 kcal mol^{-1} (tripeptide + $(TiO_2)_{10}$—alendronate → $(TiO_2)_{10}$—alendronate—tripeptide).

The advantages of forming a $(TiO_2)_{10}$—tripeptide—alendronate structure should be highlighted in comparison to forming a $(TiO_2)_{10}$—alendronate—tripeptide structure considering both modelling options. Simultaneous or supplementary addition of selected molecules during the theoretical simulations of the interactions exhibits higher values of Gibbs energies for $(TiO_2)_{10}$—tripeptide—alendronate, $\Delta_r G^*_{simult.binding}$ = -8.25 kcal mol^{-1}, $\Delta_r G^*_{addit.binding}$ = -1.80 kcal mol^{-1} than $(TiO_2)_{10}$—alendronate—tripeptide, $\Delta_r G^*_{simult.binding}$ = -6.03 kcal mol^{-1}; $\Delta_r G^*_{addit.binding}$ = 4.13 kcal mol^{-1}. One can conclude that the implant's surface is mainly covered with an outer inhomogeneous alendronate layer filled with tripeptide which is dominated in an inner layer part.

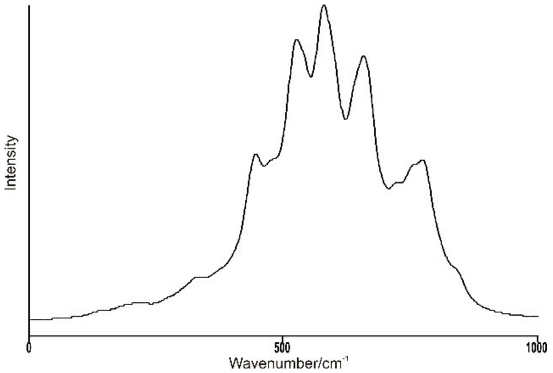

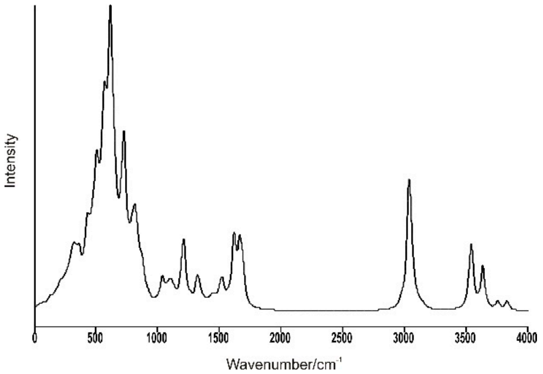

$(TiO_2)_{10}$

$(TiO_2)_{10}$–tripeptide–alendronate

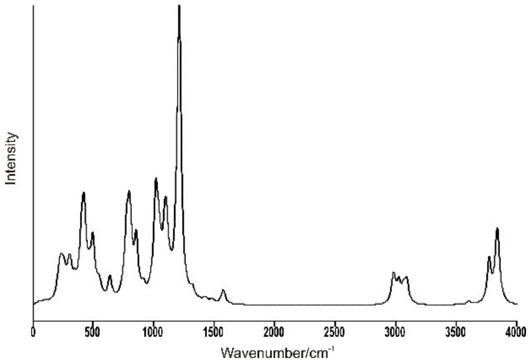

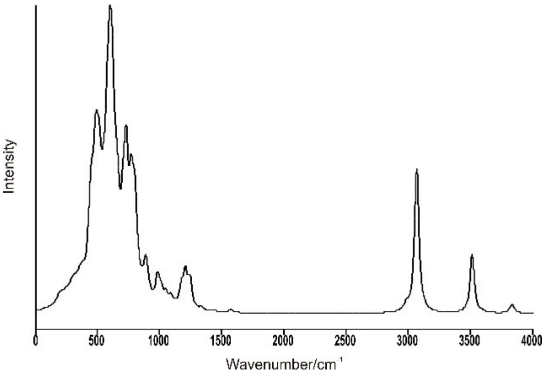

alendronic-acid-anion

$(TiO_2)_{10}$–alendronate

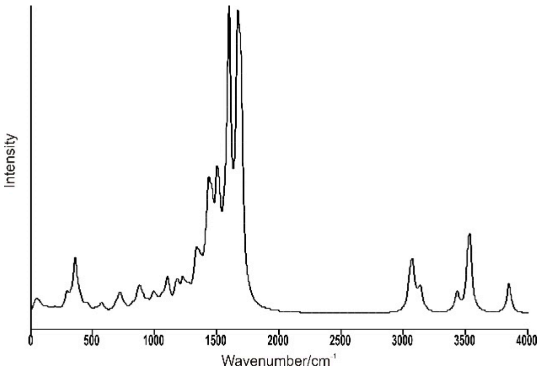

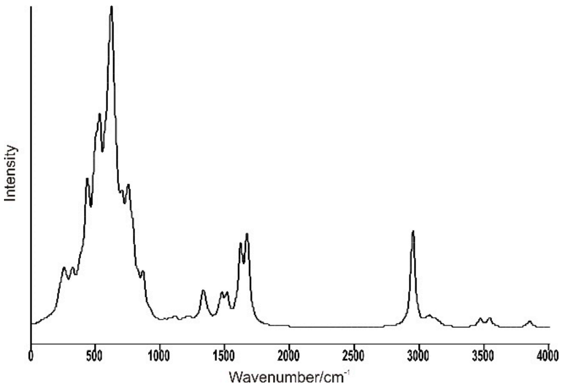

tripeptide

$(TiO_2)_{10}$–tripeptide

Figure S1: Theoretically calculated IR spectra of the investigated systems.

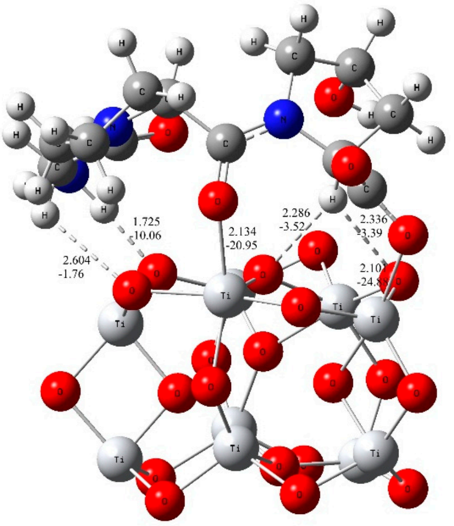

(TiO2)10−tripeptide

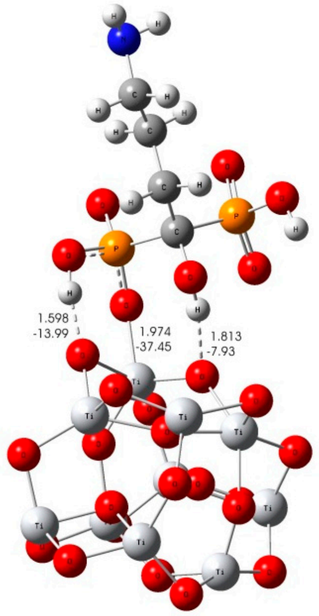

(TiO2)10−alendronate

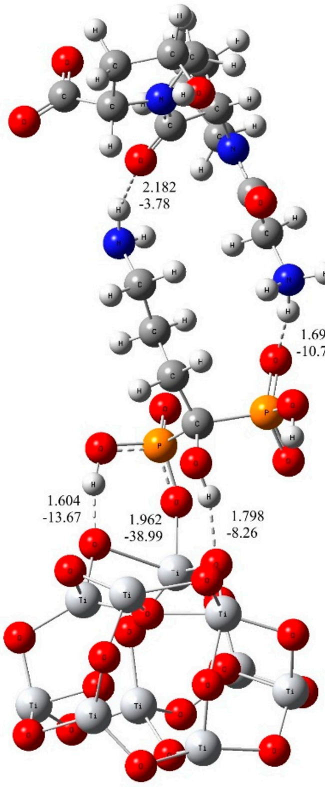

(TiO2)10−alendronate−tripeptide

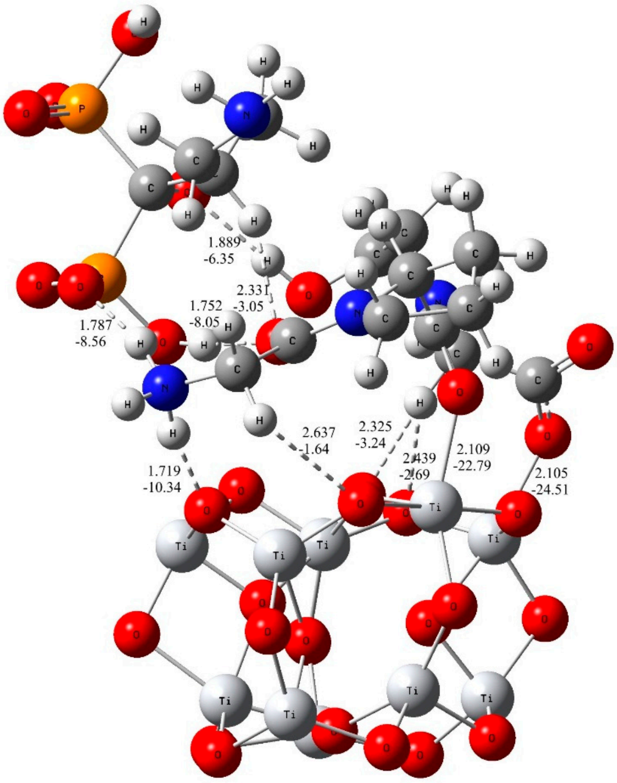

(TiO2)10−tripeptide−alendronate

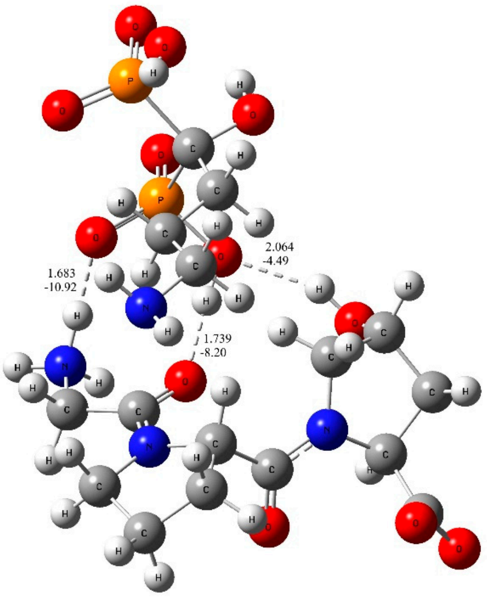

tripeptide–alendronate

Figure S2: Optimized structures of the selected systems (bond distances in Å, bond energies in kcal mol^{-1})

Table S1. Formation of single-layer and two-layer coatings. Standard state (1M) free energies of interaction $\Delta_r G^*_{INT}$ computed by using the SMD solvation model at the M06/6-311++G(2df,2pd) + LANL2DZ// M06/6-31+G(d,p) + LANL2DZ level of theory (in kcal mol^{-1}).

**Interaction**	$\Delta_r G^*_{INT}$
$(TiO_2)_{10}$ + tripeptide → $(TiO_2)_{10}$–tripeptide	-6.45
$(TiO_2)_{10}$ + alendronic-acid-anion → $(TiO_2)_{10}$–alendronate	-10.16
$(TiO_2)_{10}$ + tripeptide + alendronic-acid-anion → $(TiO_2)_{10}$–alendronate–tripeptide	-6.03
$(TiO_2)_{10}$ + tripeptide + alendronic-acid-anion → $(TiO_2)_{10}$–tripeptide–alendronate	-8.25
tripeptide + $(TiO_2)_{10}$–alendronate → $(TiO_2)_{10}$–alendronate–tripeptide	4.13
alendronic-acid-anion + $(TiO_2)_{10}$–tripeptide → $(TiO_2)_{10}$–tripeptide–alendronate	-1.80
tripeptide–alendronate + $(TiO_2)_{10}$ →	

$(TiO_2)_{10}$−alendronate−tripeptide	-7.99
tripeptide−alendronate + $(TiO_2)_{10}$ → $(TiO_2)_{10}$−tripeptide−alendronate	-10.22
tripeptide + alendronic-acid-anion → tripeptide−alendronate	1.97

Table S2. Total electronic energy, E^{Tot}_{soln}, obtained at the SMD/M06/6-311++G(2df,2pd) + LANL2DZ//SMD/M06/6-31+G(d,p) + LANL2DZ level of theory, thermal correction to the Gibbs free energy, $\Delta G^{*}_{VRT,soln}$, obtained at the SMD/M06/6-31+G(d,p) + LANL2DZ level of theory, and total free energy, G^{*}_{X}, ($G^{*}_{X} = E^{Tot}_{soln} + \Delta G^{*}_{VRT,soln}$) in water media of the investigated species (all energies in hartree).

Species	E^{Tot}_{soln}	$\Delta G^{*}_{VRT,soln}$	G^{*}_{X}
$(TiO_2)_{10}$	-2087.42891	0.01633	-2087.41258
tripeptide	-1008.91948	0.27723	-1008.64225
alendronic-acid-anion	-1423.53070	0.13691	-1423.39379
tripeptide−alendronate	-2432.47625	0.44334	-2432.03291
$(TiO_2)_{10}$−tripeptide	-3096.39435	0.32924	-3096.06511
$(TiO_2)_{10}$−alendronate	-3511.00512	0.18257	-3510.82255
$(TiO_2)_{10}$−alendronate−tripeptide	-4519.94667	0.48845	-4519.45822
$(TiO_2)_{10}$−tripeptide−alendronate	-4519.95593	0.49416	-4519.46177

Table S3. Bond lengths (d), energies (*E*) and QTAIM properties of the selected bonds in the investigated systems.

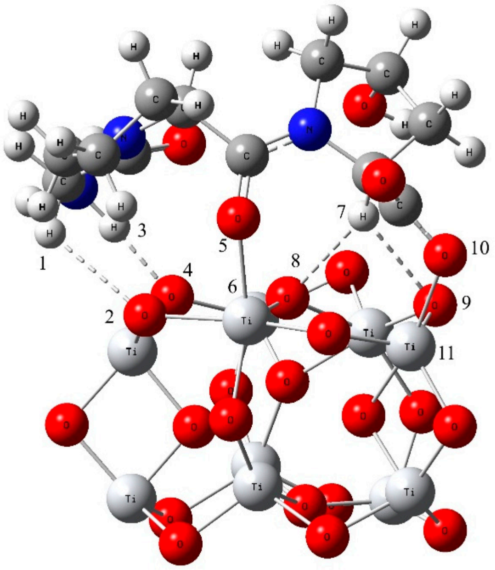

$(TiO_2)_{10}$−tripeptide

Bond	d /Å	$\rho(r_c)$/ $e\times a_0^{-3}$	$\nabla^2\rho(r_c)$/ $e\times a_0^{-5}$	$V(r_c)$/au	$G(r_c)$/au	$H(r_c)$/au[a]	E/ kcal mol^{-1} [b]
H(1)─O(2)	2.604	9.145×10^{-3}	0.0308	-0.0056	0.0067	0.0010	-1.76
H(3)─O(4)	1.725	4.149×10^{-2}	0.1263	-0.0321	0.0318	-0.0002	-10.06
O(5)─Ti(6)	2.134	5.570×10^{-2}	0.2859	-0.0667	0.0691	0.0024	-20.95
H(7)─O(8)	2.286	1.494×10^{-2}	0.0469	-0.0112	0.0114	0.0002	-3.52
H(7)─O(9)	2.336	1.470×10^{-2}	0.0466	-0.0108	0.0112	0.0004	-3.39
O(10)─Ti(11)	2.101	6.528×10^{-2}	0.3152	-0.0793	0.0791	-0.0002	-24.88

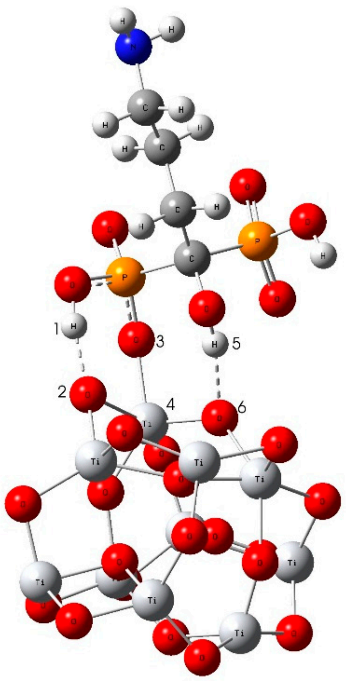

$(TiO_2)_{10}$−alendronate(**2**)

Bond	d /Å	$\rho(r_c)$/ $e\times a_0^{-3}$	$\nabla^2\rho(r_c)$/ $e\times a_0^{-5}$	$V(r_c)$/au	$G(r_c)$/au	$H(r_c)$/au[a]	E/ kcal mol^{-1}
H(1)─O(2)	1.598	5.315×10^{-2}	0.1690	-0.0446	0.0434	-0.0012	-13.99
O(3)─Ti(4)	1.974	8.723×10^{-2}	0.4894	-0.1194	0.1209	0.0015	-37.45
H(5)─O(6)	1.813	3.316×10^{-2}	0.1022	-0.0253	0.0254	0.0002	-7.93

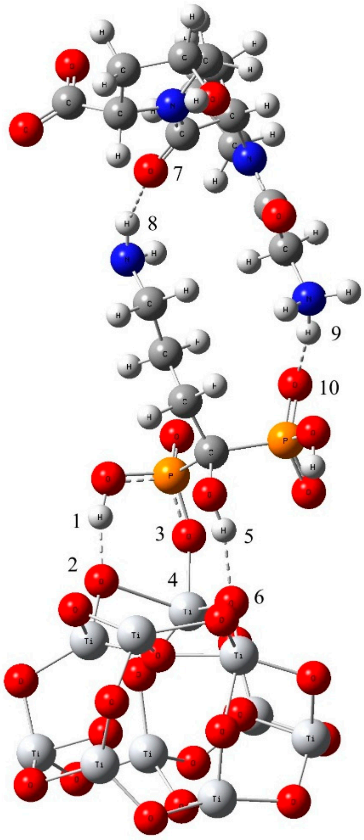

(TiO2)10−alendronate−tripeptide

Bond	d /Å	$\rho(r_c)$/ $e\times a_0^{-3}$	$\nabla^2\rho(r_c)$/ $e\times a_0^{-5}$	$V(r_c)$/au	$G(r_c)$/au	$H(r_c)$/au	E/ kcal mol^{-1}
H(1)─O(2)	1.604	5.268×10^{-2}	0.1637	-0.0436	0.0423	-0.0013	-13.67
O(3)─Ti(4)	1.962	9.089×10^{-2}	0.4854	-0.1243	0.1228	-0.0015	-38.99
H(5)─O(6)	1.798	3.437×10^{-2}	0.1069	-0.0263	0.0265	0.0002	-8.26
O(7)─H(8)	2.182	1.547×10^{-2}	0.0496	-0.0121	0.0122	0.0001	-3.78
H(9)-O(10)	1.696	4.459×10^{-2}	0.1357	-0.0344	0.0342	0.0002	-10.79

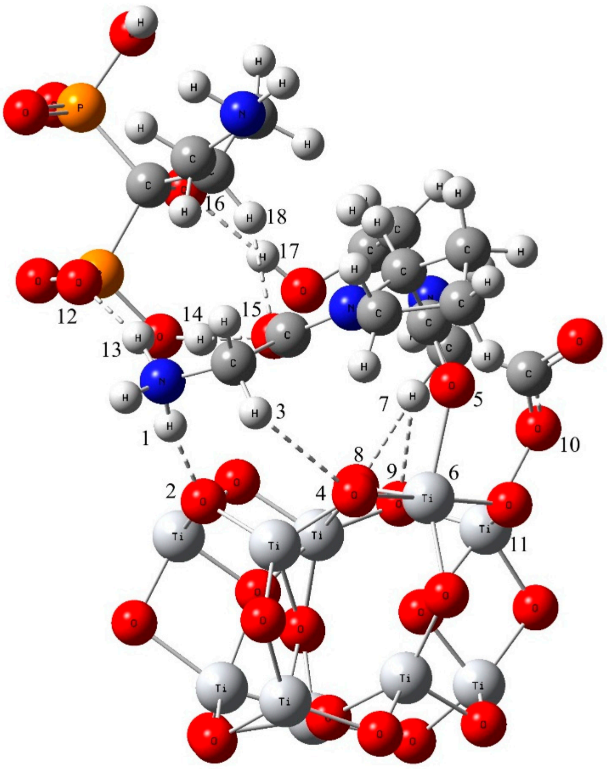

(TiO2)10−tripeptide−alendronate

Bond	d /Å	$\rho(r_c)$/ $e\times a_0^{-3}$	$\nabla^2\rho(r_c)$/ $e\times a_0^{-5}$	$V(r_c)$/au	$G(r_c)$/au	$H(r_c)$/au	E/ kcal mol^{-1}
H(1)─O(2)	1.719	4.196×10^{-2}	0.1313	-0.0329	0.0329	0.0000	-10.34
H(3)─O(4)	2.637	8.701×10^{-3}	0.0294	-0.0052	0.0063	0.0011	-1.64
O(5)─Ti(6)	2.109	5.902×10^{-2}	0.3128	-0.0726	0.0754	0.0028	-22.79
H(7)─O(8)	2.325	1.398×10^{-2}	0.0437	-0.0103	0.0106	0.0003	-3.24
H(7)─O(9)	2.439	1.226×10^{-2}	0.0394	-0.0086	0.0092	0.0006	-2.69
O(10)─Ti(11)	2.105	6.479×10^{-2}	0.3105	-0.0781	0.0779	-0.0002	-24.51
O(12)─H(13)	1.787	3.631×10^{-2}	0.1079	-0.0272	0.0271	-0.0001	-8.56
H(14)─O(15)	1.752	3.430×10^{-2}	0.1222	-0.0257	0.0281	0.0024	-8.05
O(16)─H(17)	1.889	2.727×10^{-2}	0.0823	-0.0202	0.0204	0.0002	-6.35
H(18)─O(15)	2.331	1.334×10^{-2}	0.0423	-0.0097	0.0101	0.0004	-3.05

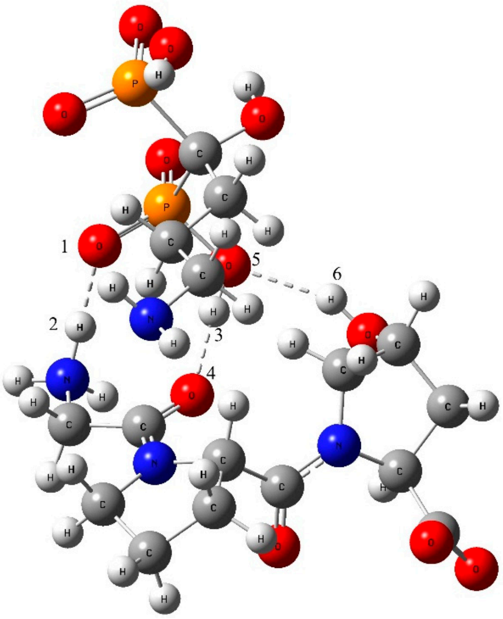

tripeptide−alendronate

Bond	d /Å	$\rho(r_c)$/ $e{\times}a_0^{-3}$	$\nabla^2\rho(r_c)$/ $e{\times}a_0^{-5}$	$V(r_c)$/au	$G(r_c)$/au	$H(r_c)$/au	E/ kcal mol^{-1}
O(1)─H(2)	1.683	$4.517{\times}10^{-2}$	0.1396	-0.0348	0.0348	0.0000	-10.92
H(3)─O(4)	1.739	$3.474{\times}10^{-2}$	0.1249	-0.0261	0.0286	0.0025	-8.20
O(5)─H(6)	2.064	$1.809{\times}10^{-2}$	0.0557	-0.0143	0.0141	-0.0002	-4.49

[a] $H(r_c) = V(r_c) + G(r_c)$; [b] $E = 0.5 \times V(r_c)$.

Cartesian coordinates of the calculated systems

$(TiO_2)_{10}$

O	0.039305	-0.015425	-0.096029
Ti	-0.142229	-0.016449	1.909261
O	1.643979	-0.081708	2.225577
Ti	2.588875	-1.632763	2.701598
O	1.385534	-2.340195	0.813656
Ti	0.500223	-1.634302	-0.700172
O	-0.835925	-2.909901	-0.425808
Ti	-0.717366	-3.425701	1.376934
O	-0.038901	-3.603219	3.256275
Ti	1.187160	-4.492811	4.427591
O	2.039784	-3.483926	5.822923
Ti	2.122282	-1.690467	5.604155
O	0.976851	-1.661306	4.177928
Ti	-0.446984	-0.773519	4.936094
O	-0.647342	0.552109	3.713348
Ti	-1.574243	-3.883261	4.129682

O	-2.165108	-4.078557	2.176736
O	0.736288	-0.670242	6.371498
O	-1.639573	-2.241342	4.926268
Ti	2.181085	-5.041926	1.774874
O	3.218363	-4.444322	0.285745
Ti	3.032921	-2.661180	-0.012552
O	2.183516	-1.956117	-1.502171
O	0.557956	-4.947496	1.108874
O	-0.982160	-1.540559	1.964775
O	-0.519545	-4.933019	5.194947
O	1.753850	-5.925654	3.534382
O	2.454572	-3.626436	2.916724
O	3.851642	-1.709565	1.363512
O	3.445548	-1.293750	4.274371

tripeptide

N	-1.185943	-0.101712	0.315797
C	-0.461936	0.906899	1.121223
C	0.935655	0.406439	1.396626
N	1.750937	1.258357	2.027904
O	1.274893	-0.730741	1.036710
H	-1.169821	-1.016582	0.778784
H	-1.012881	1.054093	2.054954
H	-0.456001	1.851413	0.571046
H	-0.750402	-0.223440	-0.603706
C	3.075815	0.833138	2.466662
C	2.961666	-0.155421	3.624727
N	4.077527	-0.815408	3.968435
O	1.893402	-0.310373	4.239215
C	3.708647	2.152533	2.921913
C	2.527102	2.906313	3.522700
C	1.365662	2.566017	2.596364
H	3.614897	0.372884	1.631824
H	4.530397	2.009019	3.629910
H	4.102515	2.677897	2.044001
H	2.324021	2.536748	4.535793
H	2.696922	3.984275	3.585184
H	0.413324	2.493816	3.132990
H	1.256641	3.301111	1.788860
C	4.106848	-1.628824	5.188188
C	3.760948	-0.840541	6.458506
O	3.261070	-1.517694	7.399023
O	4.054639	0.382983	6.502543
C	5.561356	-2.119700	5.254734

C	6.084296	-1.987758	3.831643
C	5.401074	-0.729804	3.341537
H	3.400495	-2.462292	5.089306
H	6.152213	-1.462022	5.906243
H	5.642384	-3.141252	5.638077
H	7.176671	-1.891230	3.795031
O	5.657394	-3.066189	3.003387
H	5.947471	0.156149	3.695506
H	5.332941	-0.693691	2.250193
H	5.999103	-3.889252	3.378806
H	-2.161377	0.176507	0.177131

alendronic-acid-anion

C	-0.003769	-0.046902	-0.010445
P	-0.838990	1.544174	-0.512918
O	-2.127865	1.650306	0.275468
O	0.083741	-0.033716	1.423615
C	1.443797	-0.160454	-0.489105
C	1.707612	-0.239803	-1.985748
C	3.177793	-0.488024	-2.259126
N	3.449527	-0.506996	-3.697171
P	-1.069513	-1.509859	-0.458973
O	-1.504968	-1.443287	-1.905748
O	-2.106581	-1.640104	0.638488
O	-0.065610	-2.792071	-0.243663
O	0.168322	2.673848	0.134606
O	-0.869104	1.726785	-2.015911
H	1.865262	-1.053068	-0.002001
H	1.993433	0.697570	-0.071558
H	1.115682	-1.048470	-2.444727
H	1.400485	0.690234	-2.481692
H	3.771988	0.317269	-1.806079
H	3.482559	-1.420841	-1.753139
H	-0.802369	-0.230872	1.771015
H	2.942275	-1.286431	-4.113043
H	4.435472	-0.705592	-3.848846
H	0.899671	2.884322	-0.465506
H	0.497597	-2.953087	-1.016039

tripeptide-alendronate

C	-2.183915	-6.988868	-3.566412
C	-0.956001	-6.339347	-2.929034
C	-1.175582	-5.182158	-1.966463
C	0.142929	-4.564265	-1.544090
N	-0.077115	-3.463890	-0.605901
P	-3.143415	-5.843644	-4.688827
O	-1.954673	-5.119305	-5.561900
O	-3.893274	-4.795027	-3.896613
O	-3.891721	-6.710631	-5.678520
P	-3.319098	-7.819627	-2.345665
O	-3.979801	-6.806779	-1.417202
O	-4.199347	-8.767723	-3.129141
O	-2.295633	-8.725300	-1.449059
O	-1.648406	-8.032070	-4.395382
H	-0.308978	-6.004784	-3.753908
H	-0.397962	-7.141862	-2.420350
H	-1.796308	-4.402329	-2.433886
H	-1.720909	-5.516071	-1.070691
H	0.766570	-5.323139	-1.048993
H	0.698017	-4.253042	-2.446488
H	-2.385688	-8.438113	-4.878960
H	-0.541390	-2.701370	-1.095911
H	0.818699	-3.098404	-0.291763
H	-2.155802	-8.316573	-0.564014
H	-1.571426	-4.361885	-5.093796
N	-4.448324	-7.207990	1.248582
C	-3.550439	-6.296375	1.984805
C	-2.119636	-6.692883	1.731088
N	-1.170574	-5.941869	2.288822
O	-1.848603	-7.680400	1.024848
H	-4.264286	-8.182928	1.502490
H	-3.790769	-6.337967	3.051108
H	-3.728081	-5.274525	1.632354
H	-4.304351	-7.110689	0.205786
C	0.239163	-6.166626	1.946077
C	0.689185	-7.572343	2.311574
N	1.306376	-8.292674	1.362226
O	0.552195	-7.988223	3.475338
C	0.986065	-5.101480	2.767474
C	0.000259	-4.677550	3.855555
C	-1.353777	-4.782904	3.174614
H	0.370283	-5.986484	0.874591
H	1.929055	-5.488163	3.168948
H	1.222266	-4.253443	2.113970

H	0.042501	-5.366599	4.707213
H	0.193766	-3.667411	4.226721
H	-2.177144	-4.956523	3.873351
H	-1.581995	-3.888852	2.577445
C	1.880179	-9.600714	1.687233
C	3.057848	-9.542127	2.665682
O	3.311762	-10.625305	3.262661
O	3.712477	-8.471877	2.763179
C	2.340510	-10.128996	0.322250
C	1.409120	-9.455607	-0.667501
C	1.266253	-8.058253	-0.088808
H	1.101846	-10.246245	2.115938
H	3.369656	-9.802217	0.121027
H	2.305440	-11.220239	0.262043
H	1.820182	-9.422540	-1.685223
O	0.171015	-10.158000	-0.654316
H	2.099879	-7.418941	-0.411549
H	0.319787	-7.596673	-0.387741
H	-0.480197	-9.672686	-1.189984
H	-5.424408	-7.000292	1.470801

$(TiO_2)_{10}$-tripeptide

Ti	-2.909359	-13.569328	4.964243
Ti	-0.980401	-11.197021	6.749629
Ti	-3.909927	-11.399564	6.872040
Ti	-0.621477	-8.875843	4.431242
Ti	2.115590	-10.417049	4.160303
Ti	1.738540	-12.235561	6.310638
Ti	-3.713154	-9.820138	4.545727
Ti	-0.128091	-12.068257	2.910129
Ti	-3.009074	-12.293991	2.402614
Ti	-0.176998	-14.400786	5.255802
O	1.452045	-14.051125	6.131136
O	1.586163	-11.508550	2.638160
O	-3.961111	-13.546376	3.314035
O	-4.906820	-10.096396	5.983979
O	-0.774906	-9.486101	6.253035
O	-4.162794	-13.134077	6.347788
O	-1.186535	-12.262379	1.469217
O	-2.084190	-15.134318	4.938038
O	-2.636942	-8.424394	4.548401
O	3.102102	-11.165573	5.496184
O	-2.377101	-11.241366	7.963532
O	-1.901991	-12.398445	3.776207

O	-1.206306	-13.136403	6.087365
O	-0.764018	-10.099526	3.252608
O	0.678437	-11.550337	5.019955
O	-2.722559	-11.068885	5.500799
O	-3.821071	-10.687298	2.863700
O	0.221311	-13.899108	3.615857
O	1.285760	-8.912190	4.712808
O	0.637332	-11.599578	7.634965
N	-5.278779	-8.729924	1.573270
C	-4.691519	-7.491743	2.124963
C	-3.278703	-7.335298	1.616144
N	-2.657846	-6.188898	1.926792
O	-2.751945	-8.214982	0.919356
H	-4.771287	-9.566938	1.958644
H	-4.704025	-7.564477	3.218291
H	-5.311399	-6.639288	1.831349
H	-5.204014	-8.748349	0.552773
C	-1.309238	-5.920089	1.420268
C	-0.333225	-6.979588	1.896534
N	0.497252	-7.538605	1.019130
O	-0.286317	-7.216187	3.132700
C	-0.974695	-4.547496	2.023650
C	-1.839202	-4.457179	3.278516
C	-3.123426	-5.168926	2.882248
H	-1.332029	-5.872085	0.329675
H	0.096789	-4.438545	2.223091
H	-1.264297	-3.775147	1.302303
H	-1.362416	-4.980652	4.114667
H	-2.025058	-3.423545	3.583248
H	-3.628741	-5.638415	3.732082
H	-3.829317	-4.486919	2.389244
C	1.469236	-8.581378	1.407901
C	2.595934	-8.162290	2.337406
O	3.212173	-9.160707	2.881955
O	2.923802	-6.976774	2.469161
C	2.066582	-9.033462	0.071987
C	0.936805	-8.807914	-0.911853
C	0.353789	-7.487913	-0.456187
H	0.917902	-9.406585	1.867972
H	2.920437	-8.399372	-0.203617
H	2.403660	-10.074413	0.105799
H	1.283946	-8.753679	-1.951317
O	-0.082635	-9.793353	-0.773872
H	0.924035	-6.646678	-0.865849
H	-0.692441	-7.400608	-0.755727
H	0.321059	-10.669199	-0.847739

H	-6.267786	-8.794748	1.824947

$(TiO_2)_{10}$-alendronate

C	-0.595322	-8.115197	9.756355
O	-1.224092	-9.245011	9.149281
C	-1.711844	-7.483047	10.596853
C	-1.405020	-6.261371	11.448662
C	-2.669026	-5.746086	12.108983
N	-2.376202	-4.611414	12.985622
P	0.840754	-8.739065	10.774030
O	1.655706	-9.669842	9.899222
P	-0.021312	-6.956916	8.413138
O	-1.377315	-6.426570	7.735391
O	0.071395	-9.620467	11.930101
O	1.538010	-7.620342	11.514294
O	0.778193	-5.801800	8.946914
O	0.716462	-7.812768	7.340068
Ti	0.375249	-9.255684	6.037605
O	2.119148	-9.467234	5.455164
Ti	2.348116	-10.941256	4.349560
O	0.889421	-11.929363	4.888413
Ti	-0.261264	-10.931590	2.930720
O	-0.298677	-12.781322	2.632061
Ti	-0.751513	-14.149351	3.729480
O	-1.213218	-13.695656	5.335436
Ti	-0.607317	-12.398665	6.754625
O	0.954507	-13.458364	6.995759
Ti	1.818380	-13.498197	5.360478
O	3.302219	-12.448434	4.797938
Ti	-3.443836	-11.784568	6.601319
O	-1.904001	-11.262640	5.839791
Ti	-2.499041	-9.565048	5.052519
O	-3.345054	-9.001245	3.391814
Ti	-3.062872	-10.315740	2.194189
O	-2.322389	-11.334200	3.553960
Ti	-3.509720	-12.798200	3.493947
O	-2.586202	-14.265472	3.020649
O	-4.252148	-11.796664	2.125950
O	-4.163671	-12.803691	5.247205
O	0.184016	-10.595980	7.169150
O	-1.992055	-12.666198	7.822254
O	-4.023257	-10.138341	6.167602

O	-1.804519	-8.325326	6.036640
O	-0.498673	-9.631946	4.349471
O	1.562458	-10.652599	2.690657
O	1.094559	-14.678231	4.216605
O	-1.280240	-10.205230	1.574938
H	-2.098202	-8.284756	11.242968
H	-2.526603	-7.239419	9.898592
H	-0.668863	-6.512120	12.226890
H	-0.961347	-5.458332	10.843402
H	-3.374965	-5.412911	11.336329
H	-3.161382	-6.579853	12.640229
H	-0.614485	-9.718966	8.539929
H	-1.782181	-4.934647	13.746992
H	-3.237770	-4.290641	13.420286
H	-1.688419	-7.098603	7.054610
H	-0.258689	-10.463144	11.583507

$(TiO_2)_{10}$-alendronate-tripeptide

C	-0.467806	-10.746890	7.200361
C	-1.505149	-10.169958	8.175401
C	-1.373227	-8.714997	8.603916
C	-2.325992	-8.386280	9.735748
N	-2.262014	-6.963647	10.074255
P	1.108230	-11.246031	8.069456
O	0.572437	-12.450210	9.044473
O	1.588349	-10.177903	9.041389
O	2.078441	-11.795643	7.048523
P	-0.129215	-9.599143	5.778757
O	0.657352	-8.392605	6.207122
O	0.544007	-10.428926	4.646814
O	-1.586188	-9.192107	5.241497
O	-1.064984	-11.940893	6.695821
H	-1.515061	-10.817228	9.067481
H	-2.483347	-10.307367	7.691403
H	-0.344782	-8.494940	8.927280
H	-1.584451	-8.047108	7.757230
H	-3.356107	-8.632381	9.440777
H	-2.097863	-9.043298	10.597437
H	-0.504803	-12.374738	6.012796
H	-1.283482	-6.712280	10.216119
H	-2.709321	-6.827310	10.980783
H	-1.870445	-9.835197	4.520526
H	0.303245	-13.236995	8.546356
Ti	-3.674248	-15.448581	0.854953

Ti	-0.771844	-15.049046	4.115630
Ti	-3.608363	-14.434949	3.962325
Ti	0.210722	-11.906065	3.398610
Ti	2.183588	-13.591637	1.710565
Ti	1.653853	-16.148578	2.721483
Ti	-2.663569	-12.215429	2.413525
Ti	-0.425792	-13.581971	0.291725
Ti	-3.227400	-12.966121	-0.444805
Ti	-0.916041	-16.799732	1.090486
O	0.930032	-17.328612	1.577610
O	1.397930	-13.302980	0.051662
O	-4.416676	-14.447045	-0.513044
O	-4.187784	-12.788722	3.528608
O	0.019489	-13.246361	4.530155
O	-4.328199	-15.454072	2.608211
O	-1.444768	-12.855611	-1.064057
O	-2.750730	-16.915853	0.381655
O	-1.969046	-10.975707	3.397645
O	3.137692	-15.098815	2.158943
O	-2.156582	-15.316579	5.183260
O	-2.486917	-13.984581	0.914966
O	-1.377745	-16.346037	2.696442
O	-0.663201	-12.282327	1.710476
O	0.724894	-14.579744	2.249418
O	-2.068528	-13.913021	3.200797
O	-3.509582	-11.651626	0.752820
O	-0.463205	-15.431703	-0.006934
O	1.954621	-12.117615	2.816169
O	0.789980	-16.108745	4.356764
N	0.450181	-10.341268	11.529370
C	0.350922	-8.962394	12.044226
C	-0.715503	-8.885228	13.111897
N	-0.908124	-7.685994	13.672940
O	-1.358822	-9.896283	13.435945
H	-0.487390	-10.724340	11.363161
H	0.113113	-8.303927	11.200168
H	1.326008	-8.657263	12.436196
H	0.909417	-10.960128	12.201684
C	-1.940983	-7.500835	14.688072
C	-3.337175	-7.647572	14.095468
N	-4.336847	-7.889273	14.958464
O	-3.547258	-7.500441	12.880030
C	-1.702510	-6.058436	15.157867
C	-1.162433	-5.367025	13.910046
C	-0.257933	-6.419734	13.286696
H	-1.795642	-8.228152	15.493841

H	-2.612781	-5.592730	15.549861
H	-0.948591	-6.063649	15.953827
H	-1.983749	-5.120052	13.223925
H	-0.621232	-4.444310	14.136441
H	-0.185747	-6.330038	12.197450
H	0.757133	-6.381758	13.704882
C	-5.730790	-7.802958	14.510351
C	-6.082080	-6.451884	13.871343
O	-6.984573	-6.480645	12.990281
O	-5.510171	-5.420547	14.313710
C	-6.533344	-7.986929	15.807589
C	-5.577689	-8.686202	16.762599
C	-4.241698	-8.066892	16.412332
H	-5.933444	-8.602380	13.786592
H	-6.793762	-7.007301	16.230096
H	-7.459315	-8.549253	15.653218
H	-5.836115	-8.513272	17.815147
O	-5.489383	-10.083862	16.498682
H	-4.126135	-7.098230	16.919674
H	-3.407313	-8.717753	16.689769
H	-6.368568	-10.470350	16.610579
H	0.967537	-10.349057	10.610864

$(TiO_2)_{10}$-tripeptide-alendronate

C	-1.646029	-6.117664	-2.029553
C	-0.994994	-5.214774	-0.984406
C	-1.804711	-4.066869	-0.391717
C	-0.888781	-2.992909	0.160162
N	-1.648805	-1.988469	0.902501
P	-2.185548	-5.207115	-3.566904
O	-1.028043	-4.056991	-3.762869
O	-3.525514	-4.538994	-3.344611
O	-1.989883	-6.161561	-4.726932
P	-3.058671	-7.144048	-1.370393
O	-4.017600	-6.270467	-0.570880
O	-3.611333	-7.981565	-2.504299
O	-2.332157	-8.191933	-0.355063
O	-0.601099	-7.021717	-2.432219
H	-0.086066	-4.808875	-1.455987
H	-0.649562	-5.862030	-0.166432
H	-2.472365	-3.615218	-1.141726
H	-2.460147	-4.428368	0.413727
H	-0.156811	-3.447066	0.844992
H	-0.304896	-2.563850	-0.673816
H	-0.853337	-7.418694	-3.283324

H	-2.306057	-1.541384	0.265478
H	-1.021820	-1.253026	1.218909
H	-1.992271	-7.757114	0.459276
H	-1.201707	-3.264118	-3.231984
Ti	-3.730105	-12.420441	4.374218
Ti	-1.158677	-11.611143	6.500042
Ti	-3.950324	-10.690772	6.797862
Ti	0.213249	-9.118357	4.846913
Ti	2.094560	-11.442506	3.953403
Ti	0.989077	-13.422027	5.674924
Ti	-3.084358	-8.787414	4.877039
Ti	-0.562246	-11.594729	2.481668
Ti	-3.348999	-10.664873	2.125246
Ti	-1.565136	-14.322526	4.162818
O	0.031236	-14.860010	5.064909
O	1.288567	-11.800584	2.300757
O	-4.706377	-11.591693	2.870819
O	-4.243667	-8.899257	6.375181
O	-0.338144	-9.993853	6.430182
O	-4.827421	-12.014912	5.875958
O	-1.443382	-11.026462	1.076566
O	-3.557233	-14.114297	3.813703
O	-1.619563	-7.938700	5.189949
O	2.659279	-12.903146	4.874117
O	-2.501259	-11.358460	7.773703
O	-2.341149	-11.542972	3.296912
O	-2.073963	-13.046614	5.425399
O	-0.378639	-9.778403	3.367240
O	0.294922	-12.081411	4.706723
O	-2.754216	-10.578217	5.390369
O	-3.428306	-9.066253	2.991753
O	-0.935754	-13.607191	2.706097
O	1.891832	-9.927261	4.986432
O	0.123729	-12.767397	7.195641
N	-3.956227	-6.480931	2.231309
C	-3.008670	-5.752302	3.094219
C	-1.585884	-6.036133	2.681029
N	-0.632754	-5.313544	3.281150
O	-1.312452	-6.886787	1.819045
H	-3.832829	-7.511848	2.380143
H	-3.163966	-6.083189	4.128781
H	-3.220721	-4.679291	3.044825
H	-3.849196	-6.278567	1.208509
C	0.760682	-5.393543	2.829596
C	1.294146	-6.809371	2.943234
N	1.976485	-7.340351	1.930128

O	1.136004	-7.401287	4.042765
C	1.491610	-4.426824	3.775381
C	0.601771	-4.350221	5.013497
C	-0.804443	-4.425717	4.442635
H	0.821941	-5.042349	1.796557
H	2.512846	-4.757991	3.991151
H	1.549392	-3.446663	3.286469
H	0.783397	-5.205898	5.674297
H	0.762223	-3.433796	5.587902
H	-1.531398	-4.848514	5.143658
H	-1.164535	-3.440467	4.114286
C	2.431257	-8.747988	1.943796
C	3.546388	-9.105202	2.916455
O	3.693936	-10.380012	3.091799
O	4.283924	-8.239501	3.402861
C	2.928950	-8.989457	0.512918
C	2.111577	-8.024325	-0.317979
C	2.089254	-6.785719	0.562977
H	1.562642	-9.381926	2.150878
H	3.992720	-8.730758	0.429083
H	2.793790	-10.032188	0.211007
H	2.572848	-7.804278	-1.289780
O	0.816169	-8.576483	-0.487422
H	3.025080	-6.221507	0.463941
H	1.248042	-6.135838	0.318565
H	0.309421	-8.021701	-1.113781
H	-4.913119	-6.235684	2.494730

C) Electrochemical impedance spectroscopy – Nyquist plots of experimental data

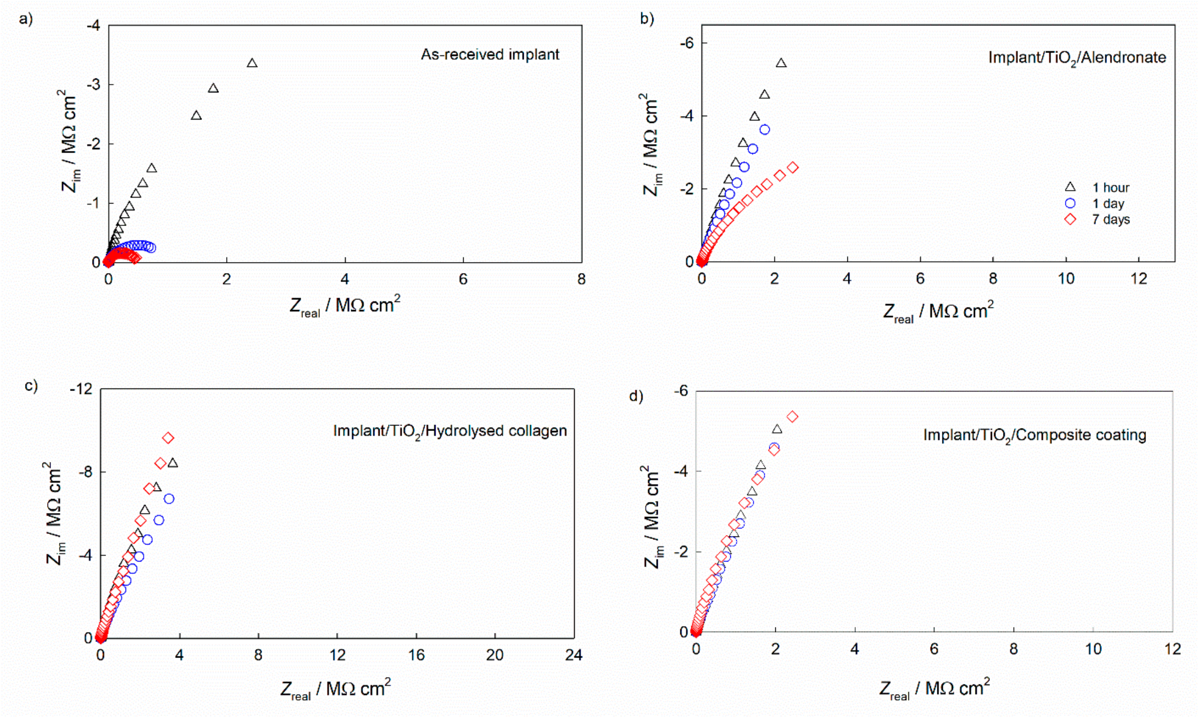

Figure S3. Nyquist plots of (a) as-received implant, (b) Implant/TiO_2/Alendronate coating, (c) Implant/TiO_2/Hydrolysed collagen coating and (d) Implant/TiO_2/Composite coating recorded after a stabilisation time of 1 hour, 1 day, and 7 days at the open circuit potential in the artificial saliva solution, pH=6.8.
